# Automatic discovery of cross-family sequence features associated with protein function

**DOI:** 10.1186/1471-2105-7-16

**Published:** 2006-01-12

**Authors:** Markus Brameier, Josien Haan, Andrea Krings, Robert M MacCallum

**Affiliations:** 1 Stockholm Bioinformatics Center, Stockholm University, 106 91 Stockholm, Sweden; 2 Bioinformatics Research Center, University of Aarhus, 8000 Aarhus C, Denmark; 3 Division of Cell and Molecular Biology, Imperial College London, London SW7 2AZ, UK

## Abstract

**Background:**

Methods for predicting protein function directly from amino acid sequences are useful tools in the study of uncharacterised protein families and in comparative genomics. Until now, this problem has been approached using machine learning techniques that attempt to predict membership, or otherwise, to predefined functional categories or subcellular locations. A potential drawback of this approach is that the human-designated functional classes may not accurately reflect the underlying biology, and consequently important sequence-to-function relationships may be missed.

**Results:**

We show that a self-supervised data mining approach is able to find relationships between sequence features and functional annotations. No preconceived ideas about functional categories are required, and the training data is simply a set of protein sequences and their UniProt/Swiss-Prot annotations. The main technical aspect of the approach is the co-evolution of amino acid-based regular expressions and keyword-based logical expressions with genetic programming. Our experiments on a strictly non-redundant set of eukaryotic proteins reveal that the strongest and most easily detected sequence-to-function relationships are concerned with targeting to various cellular compartments, which is an area already well studied both experimentally and computationally. Of more interest are a number of broad functional roles which can also be correlated with sequence features. These include inhibition, biosynthesis, transcription and defence against bacteria. Despite substantial overlaps between these functions and their corresponding cellular compartments, we find clear differences in the sequence motifs used to predict some of these functions. For example, the presence of polyglutamine repeats appears to be linked more strongly to the "transcription" function than to the general "nuclear" function/location.

**Conclusion:**

We have developed a novel and useful approach for knowledge discovery in annotated sequence data. The technique is able to identify functionally important sequence features and does not require expert knowledge. By viewing protein function from a sequence perspective, the approach is also suitable for discovering unexpected links between biological processes, such as the recently discovered role of ubiquitination in transcription.

## Background

Accurate descriptions of protein function usually arise through repeated cycles of laboratory experiments and publication, followed by expert annotation by database curators (e.g. Swiss-Prot [1] and Pfam [2]). This is, of course, a time consuming process. Computational sequence comparison methods are then typically applied to extend these annotations to related proteins from the same or a different organism. If adequate precautions are taken [3,4], this annotation transfer rapidly brings added value to what would otherwise be a large collection of unannotated sequences. Unfortunately, a substantial proportion of proteins from fully sequenced organisms remain unannotated after the application of manual and automated annotation methods; for the human proteome this fraction is approximately 40% (data from GOA Human release 28.0 [5]). Furthermore, many of the existing annotations are only partial, and one must also remember that proteins can have more than one function.

High-throughput technologies are helping to provide additional sources of information that can be used to predict protein function, typically through the detection of physical protein-protein interactions, or the analysis of gene expression patterns. Ultimately, however, a protein's amino acid sequence dictates its behaviour once it has been synthesised, and so methods for deducing function directly from sequence are needed. Alignment-based sequence comparison methods have already been mentioned as a suitable approach, but these have limited use at large evolutionary distances where annotation transfer can be unreliable. It should also be noted that alignment techniques generally require the conservation of whole domains and are tuned for optimal performance on water-soluble globular proteins. Structure-based function prediction (using predicted 3D structures) also places an emphasis on whole globular domains.

Many aspects of protein function have been attributed to sequence features that are generally found outside globular domains, including signals for subcellular targeting, degradation, calmodulin binding and post-translational modifications [6,7]. Recently, disordered regions of proteins have been receiving more attention and are no longer considered functionally inert [8]. These observations highlight the need for computational techniques that can link short regions of sequence and/or the global properties of proteins directly to function, without recourse to alignments or domain family databases.

So far, only a few researchers have begun to address this problem [9-11]. Both used a set of precalculated sequence features to describe each protein in their dataset. These features were then used to predict membership, or otherwise, to predefined functional classes. In King *et al*. [9], the features included single amino acid and dipeptide frequencies, protein molecular weight, aliphatic index, hydropathicity and predicted secondary structure. Annotation keywords for a protein and its homologues were also incoporated into the classification scheme. The target functional classes were taken from a hierarchical scheme used at that time for genome annotation by the Sanger Centre. Jensen *et al*. omitted the amino acid frequency and keyword information but additionally included predictions of various post-translational modifications, transmembrane helices and protein targeting [10,11]. The initial study by Jensen *et al*. [10] attempted to predict a set of 14 general functional classes proposed by TIGR and the six enzyme classes of the EC scheme. Their subsequent study [11] used 347 categories from the Gene Ontology [12] as targets, and found that reasonable predictions could be made for 14 of them. It is important to note that neither of these studies involved the discovery of novel sequence motifs/features more extensive than adjacent amino acid pairs.

In this study we address the issues of sequence feature/motif discovery and functional categorisation simultaneously. This is achieved using a co-evolutionary algorithm which produces two types of protein classifiers. The first classifier is fed with a single amino acid sequence and predicts membership of a functional category which has been assigned by the second classifier on the basis of Swiss-Prot annotation words. The sequence classifier makes use of one or more evolved regular expressions which are used to detect the presence or absence of sequence motifs. The annotation classifier simply uses Boolean logic to combine the presence or absence of certain words in the annotation. We call this a "self-supervised" data mining approach in which a moving target is used to train the sequence classifiers. This is in contrast to standard supervised learning approaches where the target is fixed and predetermined, and unsupervised learning where no targets are involved.

The results we obtain reinforce the widely held view that sequences hold intrinsic information about subcellar localisation [7] since we find the strongest correlations between sequence features and annotation words that describe subcellar compartments. We also find that sequence features can be linked to some general functions, such as biosynthesis and transcription, which cannot be completely explained by large overlaps with the cellular compartments in which they occur (e.g. transcription in the nucleus). The behaviour of the predictors we obtain can be analysed and the sequence features associated with various functions are presented. Finally, future development and applications of this new approach are discussed.

## Results and discussion

### Discovery of sequence-to-function relationships

**Figure 1:**
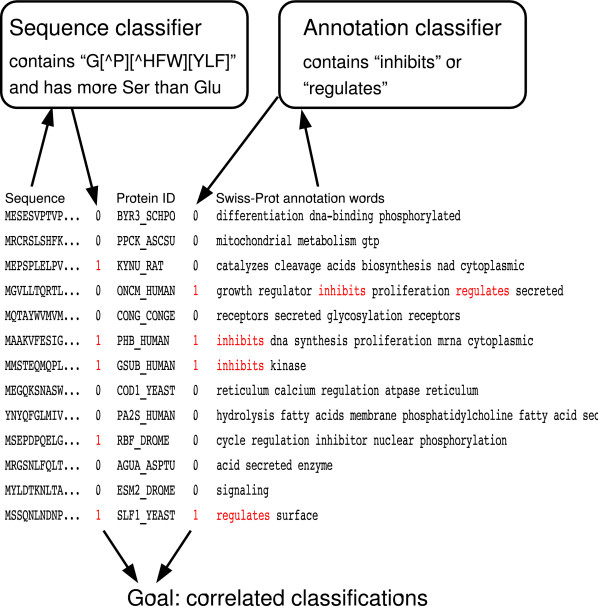
**Outline of approach: simultaneous sequence and annotation classifications**. Part of the dataset is shown with sequences (to the left) and Swiss-Prot annotation words (to the right). The evolutionary search produces two independent classifiers which act on the two types of information. Fictional examples of these classifiers are shown. Two binary vectors are produced from the application of these classifiers to their respective inputs. Ideally, a pair of classifiers would produce identical (non-trivial) binary vectors. The goal of the evolutionary search is to maximise the correlation between these vectors.

**Figure 2:**
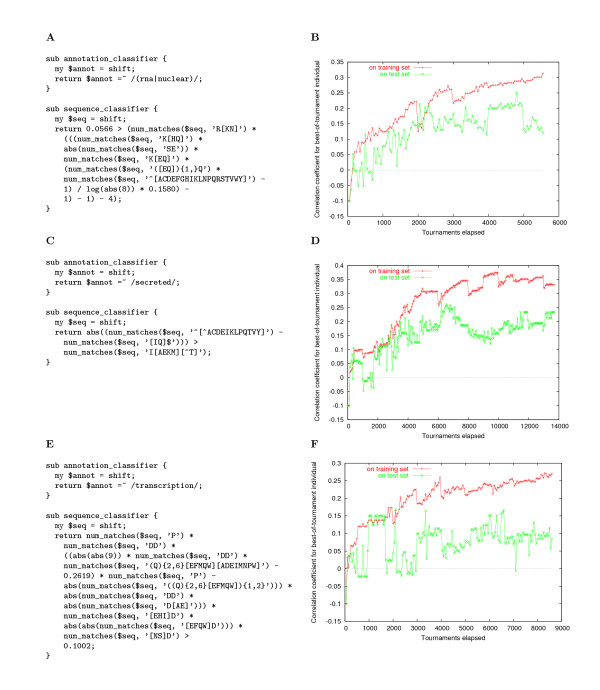
**Examples of sequence-to-function relationships found by self-supervised learning**. Three examples of sequence classifiers and their associated, co-evolved annotation-based classifiers are shown in panels A,C&E. In panels B,D&F, the correlation between the sequence-based classification and the annotation-based classification is shown for both training and testing data during the 8 h runs which produced the final individuals shown in panels A,C&E. Although these are hand-picked examples, note how the test set correlation generally follows the training set correlation in an upward trend. Because the test set proteins are minimally related to the training set proteins (less than 10% sequence identity), this shows that general sequence features related to function have been discovered.

Briefly (see Methods for full details), a non-redundant set of 2146 proteins was generated such that no two sequences share more than 10% sequence identity. Each protein is tagged with one or more words from its UniProt/Swiss-Prot annotation, hereafter referred to as "annotation words". The data is split into training and testing sets of 1609 and 537 proteins respectively. Our "self-supervised" evolutionary learning approach is then applied to find sequence-to-function relationships. It does this by simultaneously searching for sequence- and annotation-based classification rules which overlap as far as possible on the training set proteins, as illustrated in Figure 1. Figure 2 shows the outcome and progress of a few hand-picked runs. An example of an evolved sequence-to-function predictor is given in Figure 2(A). This predictor has found a correlation between the annotation of a protein with the words "rna" or "nuclear" (see annotation_classifier) and an arithmetic inequality based on sequence pattern frequencies (see sequence_classif ier). The evolved predictor shown in Figure 2(C) has "chosen" a single annotation word target ("secreted"), and the sequence classifier again uses several sequence patterns, including one three-residue pattern (I [AEKM] [^T], which means "I followed by A, E, K or M followed by anything except T").

The plots in Figure 2(B&D) show the progression of the correlation between the functional class predicted from sequence and the functional class assigned based on annotation words as the evolutionary search proceeds. The performance on the test set proteins tends to lag behind the training set performance, however it does usually follow an upward trend. The averaged results from 250 independent runs (see Methods for more details) are shown in the upper part of Table 1. The mean correlation coefficient between predicted and real functional class for the test set proteins is only 0.112 but this is significantly greater than the correlations obtained from two control experiments (two-tailed unpaired Student's *t*-test for two means; *P <*0.001). In one control, sequences are shuffled with respect to their annotation words (i.e. each sequence is assigned the annotation words belonging to another protein). In the second control, each amino acid sequence is shuffled in a residue-wise manner prior to training (while the annotation words remain unchanged). In our previous work [13], where we used a similar approach to discover sequence features associated with the nuclear localisation of proteins, the mean correlation coefficient obtained from single predictors was 0.29 (and jury predictors performed even better). Why is the performance with the new self-supervised method so much worse? In this work, we made two major changes to the approach, which are described below.

Firstly, during dataset construction, there are no special quality controls imposed on negative examples (proteins *not *annotated with particular word). In our previous work, the non-nuclear proteins in our training and testing sets had to have some positive annotation for another cellular compartment, which helps eliminate proteins whose nuclear localisation status is unknown. This is standard practice in protein function prediction (see ref. [10], for example). Therefore the datasets used in this study have a lower contrast between positive and negative examples, and lower prediction accuracies are expected. Indeed, when the methods used in this work are applied to the higher-contrast dataset in ref. [13], the mean correlation coefficient rises to 0.20 (full data not shown).

The second change in our approach is that the search algorithm is encouraged to find sequence-order dependent features (see Methods for more details). This actually decreases the performance of the evolved predictors, but we are prepared to accept this in order to find potentially interesting sequence motifs. When the mechanism encouraging sequence-order dependent feature discovery is switched off, the mean correlation coefficient rises to 0.28 (full data not shown) when using the higher-contrast training data as described above. This is close to the 0.29 correlation obtained in ref. [13], indicating that the two methodological changes account for the lower prediction performances presented here.

Function predictors obtained directly from our self-supervised approach unfortunately do not have either the specificity (around 28%) or sensitivity (around 23%) to be useful to biologists. However, the value of our approach lies in the *discovery *of potentially novel sequence-to-function relationships. At a later stage, more accurate predictors can be obtained by removing the sequence-order dependency, by the use of jury predictors, and by constructing higher contrast datasets with the help of expert biological knowledge to define both positive and negative examples.

So which sequence-to-function relationships are discovered with this technique? The most common annotation words used to form functional categories are also shown in the upper half of Table 1. The cellular compartments dominate this list, as would be expected from previous studies which have explored the relationship between sequence and subcellular localisation [14-16].

#### Beyond cellular compartments

To explore the relationship between sequence and more specific functions we performed another 250 runs where the major cellular compartment words had been removed from the vocabulary used to generate annotation classifiers. Two other small changes were made to the protocol as detailed in Methods. One hand-picked predictor is shown in Figure 2(E). One of the longer and perhaps more interesting regular expressions is (Q) {2,6} [EFMQW] [ADEIMNPW], which can be interpreted as "matches between 2 and 6 Q's followed by E, F, M, Q or W followed by A, D, E, I, M, N, P or W". Patterns similar to this will be discussed in the context of transcription later in this paper. The corresponding performance plot in Figure 2(F) shows the test set correlation rising (noisily) as the run progresses. The mean correlations for these experiments are shown in the lower half of Table 1. There is a larger gap between the training and testing correlations which indicates more overfitting but, as with the first set of 250 runs, the mean test set correlation (0.0603) is significantly greater than either of the two controls (two-tailed unpaired Student's *t*-test for two means; *P <*0.001). The results indicate that some aspects of function are encoded in sequence features that are detectable and generalisable with this approach. The 10 most common annotation words used to define functional categories are listed in the lower half of Table 1. The relationship between the top three words: "inhibits", "biosynthesis" and "transcription" and the compartments "secreted", "cytoplasmic" and "nuclear" will be discussed in a following section.

#### Intra-family motifs

If our training and testing datasets are constructed with a less strict sequence identity cutoff, our algorithm is able to discover motifs that are present across multiple sequence family members. For example, using a 50% cutoff we find that the H-R-D motif is frequently associated with the term "kinase" (data not shown). Encouragingly, the kinase H-R-D motif has been mentioned several times in the literature [17,18]. However, intra-family motifs are not the subject of this study, and a strict 10% sequence identity cutoff is used throughout.

#### The predictor map

**Figure 3:**
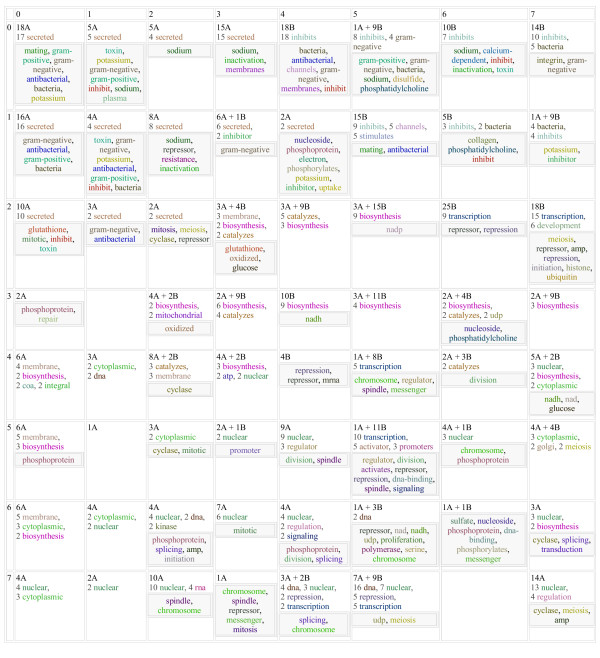
**Predictor map**. A total of 500 sequence-to-function predictors were produced by the self-supervised genetic programming approach. In this figure, an 8 × 8 Self-Organizing Map (SOM) is used to cluster the predictors based on the pattern of sequence-based test set classifications. Predictors which classify similar subsets of the sequences will be localised to the same region of the map. Each SOM node is annotated as follows (the example used is at row 3 column 2): the number of "A-type" and "B-type" predictors which map to this node (e.g. "4A + 2B"); the common target words for the annotation-based classifier and their frequencies (e.g. "2 biosynthesis, 2 mitochondrial"); the inset boxes show which annotation words are over-represented in the test set sequences which are positively classified by the sequence-based classifier (e.g. "oxidised"). See Methods for detailed information.

We next analyse the behaviour of all 500 self-supervised predictors (250 "A-type" which used all annotation words and 250 "B-type" which used only non-compartment words) by comparing the binary outputs of their evolved sequence_classifier subroutines on the 537 test set sequences. Thus each predictor is represented by a 537 element binary vector, and these are clustered using Kohonen's Self-Organizing Map (SOM) [19]. The SOM is a competitive spatial clustering technique which effectively "flattens" high-dimensional data onto the low-dimensional grid, preserving relationships in the input data as far as possible. The aim of the clustering is to group together predictors which produce positive predictions for similar subsets of the test set. Figure 3 shows the 500 predictors projected onto an 8 × 8 SOM. The number of "A-" and "B-type" predictors which cluster to each grid node are shown in black text. Below this, the frequently occuring annotation words in the annotation_classifier subroutines are shown in coloured text (recall that these define the "target function" of the predictors). The non-random distribution of the annotation words is clear, for example many "secreted" predictors map to the upper left corner of the map, "inhibits" predictors to the upper right corner, and so on. While this clustering conveniently summarises the different predictor behaviours, it is actually more informative to study the frequencies of the annotation words belonging to the *test set sequences *which are positively predicted by the predictors in each cluster. Therefore Figure 3 also shows shaded inset boxes which list the annotation words whose observed frequency in positively predicted proteins is three or more times the expected background frequency (see Methods for full details).

We now discuss an example to aid the interpretation of Figure 3. The grid node located in row two, column seven has 18 predictors allocated to it. A majority (15/18) use the word "transcription" in their annotation_classifier subroutine, and six of the 18 use the word "development". These two words are usually present in the same logical expression "transcription OR development" (it occurs 5 times, see Additional file 1). The corresponding over-represented test set words (shaded inset) include "repressor", "repression" and several other DNA-related words. Transcription is well known to involve activator and repressor proteins/domains and the development of multicellular organisms is largely controlled at the level of transcription. Therefore the over-representation of "repressor" and "repression" indicates that these 18 predictors are correctly predicting involvement in transcription or development for some of the previously unseen test set proteins. Interestingly, "ubiquitin" is also over-represented in the positive test set sequences. We have since found evidence in the literature that ubiquitination is indeed important in transcription [20].

Note that although the more common annotation words (e.g. "secreted", "nuclear", "transcription", ...) do not appear in the shaded inset boxes of Figure 3, there is still generally good agreement between target annotation words and over-represented test set annotation words for each cluster of predictors.

### Prediction of function vs. compartment

#### Distinct positioning on predictor map

Do our self-supervised function predictors actually predict specific functions (e.g. "transcription")? Or are they simply predicting targetting to a subcellular compartment in which a particular function is predominant (e.g. "nuclear")? This question can in part be answered by studying Figure 3. For instance, there is a cluster of "transcription" predictors at row two, columns 6–7 which is distinct from the large group of "nuclear" predictors at the bottom of the map.  This indicates that a different set of proteins are positively predicted by these two different clusters of predictors. Likewise, there is a separation between the "secreted" predictors (top left) and the "inhibits" predictors (top right). On the whole we see separation between the "A-type" runs (all annotation words) and the "B-type" runs (excluding location words), but this could be a consequence of the minor differences in the protocols of these runs (see Methods). Therefore we chose to produce a set of fixed-target function predictors under identical conditions so that fair comparisons can be made.

#### Fixed-target function predictors

**Figure 4:**
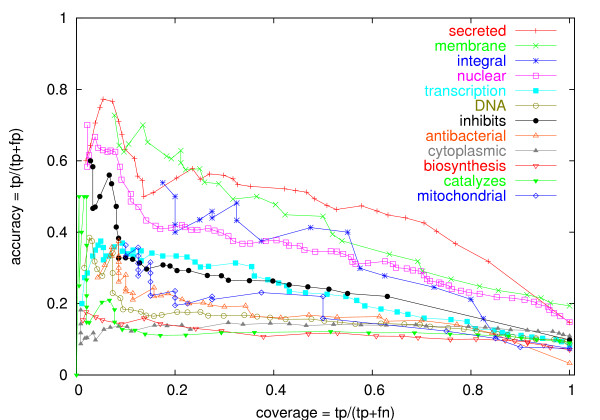
**Performance of fixed-target function predictors**. Twelve function predictors were evolved with fixed targets (listed in the Methods) instead of the evolved targets used in the self-supervised approach. Each predictor is constructed from 100 independently evolved classifiers, and its "prediction score" is simply the fraction of positive classifications (out of 100). Accuracy and coverage are presented at all possible prediction score thresholds (stopping when the number of positive predictions falls below 10). In the accuracy and coverage calculations: tp = true positives, tn = true negatives, fp = false positives, fn = false negatives.

A fixed-target predictor is produced with the standard supervised learning approach and is implemented simply by hard-coding the annotation_classifier subroutine with one or more chosen annotation words. To illustrate this, the hard-coded subroutine for a "secreted" predictor would look identical to the evolved subroutine shown in Figure 1(C). The targets were chosen manually by examining the predictor map (Figure 3) for the functions most commonly used by the self-supervised approach (such as "secreted", "inhibits", "antibacterial", "biosynthesis", "transcription", and so on). For each target function, a combined jury predictor is made using the outputs of 100 independently evolved classifiers. The *consensus prediction score *for a given sequence is simply the fraction of the classifiers which "voted positive". The performance of these fixed-target predictors is summarised in Figure 4, with accuracy *vs*. coverage shown at different score thresholds. For most target functions, the accuracy increases as the threshold increases, with a corresponding decrease in coverage. This plot shows that the accuracy of prediction to cellular compartments (e.g. "secreted", "membrane", and "nuclear") is generally higher than the accuracy of function predictions (although "transcription" and "inhibits" are predicted quite well).

### Correlations between function predictors

**Figure 5:**
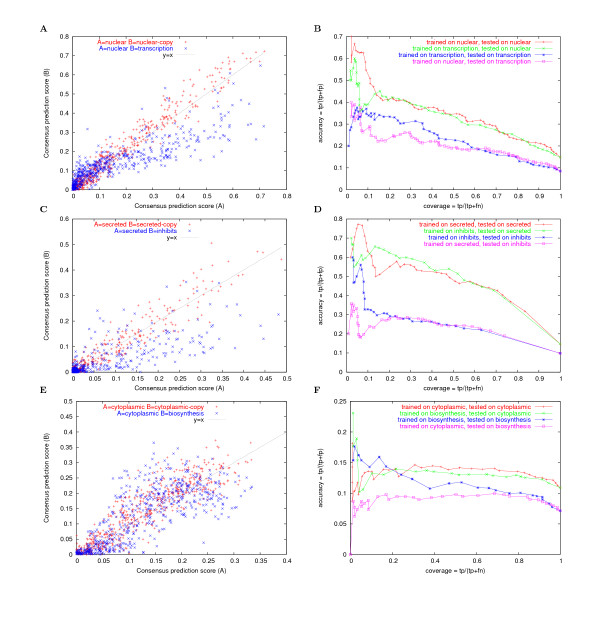
**Comparison of location *vs*. function predictors**. In panel A, the consensus prediction scores from two different fixed-target predictors over 537 test set sequences are shown in scatter plot form. The red points show scores for two identical but independently trained "nuclear" predictors. As expected, a strong correlation exists between the scores of these two predictors. The blue points show scores from a "nuclear" predictor plotted against the scores from a "transcription" predictor. The scores are still quite well correlated but the distribution of points mainly below the diagonal suggests that proteins that get high scores for "nuclear" do not always have equally high scores for "transcription", which agrees with general observations that not all nuclear proteins are involved in transcription (but all transcription proteins are nuclear). In panel B, accuracy *vs*. coverage plots are shown for the four combinations of predictors trained and/or tested on "nuclear" and/or "transcription". The data shown here are for the pooled test set proteins from a four-fold cross-validation experiment. The noteworthy result here is the increased performance of the "transcription"-trained predictor (blue line) compared to the "nuclear"-trained predictor (magenta line) when predicting "transcription". Panels C & D show the equivalent data for "secreted" *vs*. "inhibits" predictors Panels E & F show the data for "cytoplasmic" *vs*. "biosynthesis" predictors.

There are three ways to analyse the overlap between function and compartment. First we can look at consensus prediction scores produced by different predictors on the same set of proteins. Table 2 shows the correlation coefficients between the prediction scores of various pairs of predictors over the 537 test set proteins. To give an idea what the maximum expected correlation might be we have included three additional jury predictors which are copies of three selected cellular compartment predictors (but are evolved independently). These three copies give the highest correlations as expected. The next-highest correlation comes from the "nuclear" *vs*. "transcription" comparison; the actual distribution of scores is shown in Figure 5(A). From this we can conclude that proteins with a good "transcription" score generally also have a good "nuclear" score, but proteins with good "nuclear" scores do not always have good "transcription" scores. This agrees with the commonsense dictum that "a protein can be nuclear without being transcription-related but it can't be transcription-related without being nuclear". These results suggest that the predictors have some ability to differentiate between transcription-related function and nuclear localisation. Clearly though there is also considerable overlap between the behaviour of the predictors.

The correlation between prediction scores for "inhibits" and "secreted" is also quite strong (see Table 2 and Figure 5(C)). Again it seems that a high score for the function is usually accompanied by a high score for the compartment. The scores for "cytoplasmic" and "biosynthesis" also correlate well (Table 2), but the distribution in Figure 5(E) indicates that biosynthetic proteins are not simply a subset of cytoplasmic proteins (there are many blue points above *and *below the *y *= *x *line).

#### Swapped training and testing targets

The second approach to determine if predictors can distinguish between function and compartment is to compare the performance of predictors trained on function but tested on compartment and *vice versa*. This is shown for the "nuclear" & "transcription" pair in Figure 5(B), again using accuracy *vs*. coverage plots (introduced in Figure 4). The plot shows that the "nuclear" prediction performance (on the test set) is roughly the same, regardless of the target used during training ("nuclear" or "transcription"). This could be expected, again from the common knowledge that "all transcription proteins are nuclear". When tested on "transcription" prediction performance, however, the predictors trained specifically for this function perform better, suggesting that sequence features specific to "transcription" have been learnt.

When comparing predictors trained and tested on the different combinations of "secreted" and "inhibits" (Figure 5(D)), we see that the function-trained predictor is better at predicting the compartment than the compartment-trained predictor when the coverage is between 0.15 and 0.5 (green *vs*. red lines). This is surprising, but could be explained by a large overlap between the function and compartment and the presence of more specific sequence features associated with the function. When tested on the ability to predict the function "inhibits", the function-trained predictor performs better than the compartment-trained predictor at low coverage (< 0.2, blue *vs*. magenta lines). However, we have to be cautious in this region of the plot because of the small number of predictions used to calculate the accuracy.

In Figure 5(F) the only predictor which has a gradually increasing accuracy curve is the one which is trained and tested on "biosynthesis" (blue line). This suggests that, with the current approach and dataset, "cytoplasmic" cannot really be predicted at all. We should stress, however, that the test set prediction accuracy for the "biosynthesis" predictor is poor (the best case accuracy is 0.15, and the background frequency or "random" prediction accuracy is 0.07). Reasons for the comparatively poor performance of our approach have already been discussed. The key observation here is that there is a detectable seqeunce-to-function signal for proteins annotated with the word "biosynthesis", which is independent from the (weaker) signals correlated to cytoplasmic localisation.

#### Sequence features

The third approach is to look in more detail at the sequence features discovered/used by the predictors. The initial step in this analysis is to determine which are the positively and negatively influencing regular expressions (sequence patterns) in an evolved sequence_classifier subroutine. In some cases this would be easy to do by eye (see Figure 2(C)), but often genetic programming produces complex expressions that are difficult to read and may behave in a non-linear fashion (see Figure 2(A&E)), for example). As described in the Methods, we estimate local derivatives for each of the regular expression terms in the evolved subroutine with respect to its output over the training set proteins. Then all the evolved regular expressions belonging to a predictor (which is made up of 100 independently evolved classifiers) are scanned against the test set sequences. Each residue in the test set is initially assigned a zero score, but each time a regular expression matches a residue, its score is updated by a positive or negative amount (for positively and negatively influencing regular expressions, respectively). Finally we extract the highest and lowest scoring sequence fragments from the sequences for further analysis.

We should point out that the sequence feature analysis does no more than summarise the correlation of sequence features to annotations in the training data. We know that our algorithm overfits the data to some extent, therefore we checked all four cuts of the training/testing data for evidence of over-fitting. Only the "stable" sequence features, discovered in all four (overlapping) training sets are presented below.

**Figure 6:**
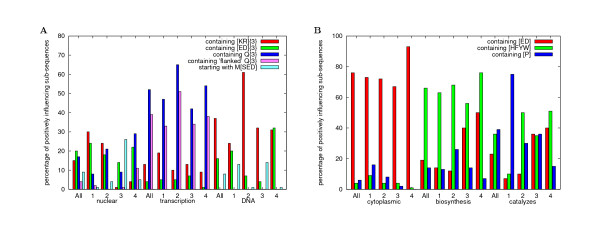
**Function-related sequence features**. The sequence_classifier subroutines of fixed-target predictors contain one or more evolved regular expressions which may influence the classifier in a positive or negatively way. As described in the Methods, this positive or negative influence can be determined with an approximation method. The positively influencing regular expressions are matched against test set sequences (cuts 1 to 4 of the data individually, or pooled together, indicated with "All" in the figure). The 500 most-matched residues or sequence fragments are then analysed manually for recurrent patterns. In panel A, we summarise the sequence features that are important for predictors of the functions: "nuclear", "transcription" and "DNA". As expected, sequence features containing multiple lysine and arginine residues are an important signal in nuclear proteins (the pattern [KR] {3} is found in approximately 15% of the top 500 positively influencing residues for "nuclear" predictors). Other signals thought to be involved in protein-protein interactions in the nucleus are also identified by this analysis: repeated acidic residues and polyglutamine. The polyglutamine feature, and particularly polyglutamine flanked by at least one of the residues D/R/H/A/N/K/S/L/E/P/T, is a stronger signal for "transcription" predictors. In panel B, the same analysis is performed for predictors of "cytoplasmic", "biosynthesis" and "catalyzes". In this case only single-residue "features" are apparent from the data. For instance, aromatic residues are more important for predictors of "biosynthesis" and "catalyzes" than for "cytoplasmic" (green bars).

The fixed-target predictors of "nuclear", "transcription" and "DNA" annotations make use of an interesting repertoire of sequence features. As expected from our knowledge of nuclear import signals [13,21], all three predictor types look for tracts of lysine and/or arginine (K/R) typically 3 to 5 residues in length. These K/R features seem to be most important for the "DNA" predictor (red bars in Figure 6(A)), representing around 30% of the highest scoring sequence fragments. We also observe a dependence on tracts of negatively charged glutamate and aspartate (E/D) residues for predictors of "nuclear" and "DNA" (and to a lesser extent, "transcription", see the green bars in Figure 6(A)). Acidic domains similar to this have been associated with protein-protein interactions in the nucleus [22-24], in addition to various non-nuclear roles [25-27].

The most clear-cut distribution can be seen for polyglutamine-containing (polyQ) features. These are most important in the "transcription" predictor (around 50% of high scoring fragments), moderately important in the "nuclear" predictor and not at all important for the "DNA" predictor (dark blue bars in Figure 6(A)). Poly-Q tracts are known to be involved in transcriptional activation (see ref. [28] for a review). Transcriptional activation domains are generally involved in stabilising/assembling the transcriptional machinery (as opposed to the DNA-recognition process). The molecular details of polyQ interactions are unclear, despite considerable interest in neurodegenerative diseases, such as Huntington's disease, in which mutant polyQ-expanded proteins are associated with late-onset neuronal death.

Closer manual inspection of the high scoring sequence fragments from the "transcription" predictor showed that the polyglutamine tract tends to be flanked by the following residues (with a convenient mnemonic): D, R, H, A, N, K, S, L, E, P, T. The flanked polyglutamine feature is very strongly associated with the "transcription" predictor (magenta bars in Figure 6(A)). These flanking residues tend to have either small or charged sidechains, with the exception of leucine which is bulky and hydrophobic. The biological significance of the flanking amino acids is not yet clear.

An interesting N-terminal sequence feature also appears to be important for just the "nuclear" and "DNA" predictors (cyan bars in in Figure 6(A)). This feature is characterised by a negatively charged amino acid (or serine) following the N-terminal methionine.

The predictors of "secreted", "inhibits" or "antibacterial" do not exhibit clear differences in terms of the positively influencing features. A possible positive feature associated with all three predictors is the presence of a Cys, Gly or Pro and a Lys or Arg (in any order); this feature constitutes 20% of the high scoring fragments (data not shown). There are clear differences in the negatively influencing sequence features. Lysine and glutamate have a strong negative influence on "secreted" predictors (present in 86% of low-scoring fragments, data not shown), but not on predictors for "inhibits". Predictors for "antibacterial" show that glutamate and aspartate are negatively influencing (87% of low-scoring subsequences, data not shown), but there is no such role for lysine. Negative sequence signals are difficult to explain, however, and are not discussed further.

The sequence features having a positive influence on the predictors for "cytoplasmic", "biosynthesis" and "catalyzes" are summarised in Figure 6(B). No obvious alignable motif could be identified from the high-scoring fragments, but this was not expected because most protein families have distinct 3D structures and the active sites of enzymes tend to be formed from non-contiguous residues. However, short high-scoring fragments identified by the "biosynthesis" and "catalyzes" predictors typically contained one or more aromatic residue (green bars in Figure 6(B)). Histidine is well known as an active site residue due to its ability to reversibly accept hydrogen ions and to coordinate with metal ions. Phenylalanine and tryptophan have been shown to be important in maintaining the geometry of the active site through stacking interactions with other aromatic moieties, including catalytic histidines [29-31] Acidic residues (red bars), which are also well known to participate in catalysis, are important for all three predictor types, but most prominent in the "cytoplasmic" predictor. Proline (blue bars) is linked to "biosynthesis" and "catalyzes", but is not well known as an active site constituent. In fact, according to the The IMB Jena Image Library Site Database [32], proline is ranked 19th out of the 20 amino acids according to the number of times it occurs in active sites (using all known enzyme structures in the PDB). Interestingly however, proline ranks 7th according to its occurrence in the environment surrounding known active site residues (Rolf Huehne & Juergen Suehnel, personal communication). The general agreement between our sequence-based analysis and the structure-based survey leads us to suggest that proline has an important accessory role in maintaining the geometry of active sites.

#### Are sequence-to-function relationships discovered?

We conclude that at least two functions, "transcription" and "biosynthesis", are associated with specific sequence features that are not simply a consequence of overlapping subcellular localisation. The most compelling evidence comes from the accuracy *vs*. coverage plots in Figure 5(B&F) where the training and testing targets were swapped, and Figure 6, where different contributing sequence features were found for each type of predictor. We draw further encouragement from the previously documented functions of some of the sequence features found automatically by our method (e.g. polyQ in transcription activation).

## Conclusion

Existing function prediction methods are forced to take a simplified view of protein sequences, for example by considering amino acid composition, secondary structure predictions and the presence of known motifs. The preparation of input data for these methods is therefore heavily dependent on human knowledge and expertise. In this study we show how an open-ended evolutionary algorithm can automatically discover features in unprocessed amino acid sequences that correlate with protein function. Our algorithm is also unique in the way it self-selects target functions while it learns these sequence features.

The most complex feature discovered so far is the "flanked polyglutamine tract" associated with transcription, but many other features are much less specific, for example the "containing proline" feature associated with catalysis. Our algorithm is designed so that every sequence-to-function predictor makes use of at least one sequence order-dependent feature. However, this feature can either be positively or negatively associated with the function, and may escape our attention if it is not clearly represented in the high (or low) scoring sequence fragments. Sometimes the sequence order-dependent features are a consequence of overfitting to the training data. There is clearly room for improvement so that more numerous and higher quality sequence features can be discovered for a variety of functions.

Feature discovery is currently limited by two interrelated factors: the size of the non-redundant protein dataset and the completeness of their annotations. Our dataset is relatively small because we have only accepted sequences with high-quality human annotations which contain at least one of the 150 most frequent words. We could increase the amount of annotated training data by including homology-based electronic annotations, as used by both King *et al*. [9] and Jensen *et al*. [10,11]. We also estimate that a less stringent sequence identity cutoff of 30% would increase the dataset size by around 25%, although at this level, some "pollution" by family-related functions would be introduced [3]. However, in order to make the biggest impact on dataset size and quality one might use the Gene Ontology (GO) [11,12] to describe protein function. Firstly, the combined coverage manual and electronic GO annotations is quite good (60% of human proteins and rising). Secondly, the hierarchical structure of GO provides a more consistent description of protein structure at many different levels. The word-based approach we have used is easy to follow, but it is of course limited because words are often ambiguous out of context. In contrast, the GO annotations benefit from the correct judgements of expert annotators.

The free-text UniProt/Swiss-Prot annotations we used may have one advantage, however. They contain information about ligands, cofactors, modifications and interaction partners that is not (yet) available in GO. For example, proteins targeted by ubiquitination may not be flagged as such in GO, but these are certainly suitable targets for sequence feature discovery. Further information about interaction partners could be drawn from databases such as KEGG [33] or the full-text literature.

It is also interesting to look at the evolved annotation word combinations which define the target functions. In the original set of 250 "A-type" predictors we saw 15 instances of the combination "nuclear OR DNA" and 9 instances of "nuclear OR RNA". (All the annotation word combinations are shown in Additional file 1, for the interested reader.) The association of "nuclear" with "DNA" and "RNA" is perhaps not surprising. Our algorithm has presumably learnt that if proteins are not annotated with the word "nuclear" (maybe we excluded a "by similarity" annotation) then the word "DNA" is also a good indication of nuclear localisation. Likewise, most RNA processing takes place in the nucleus. Interestingly, "ubiquitin" also co-occurs with "nuclear" quite frequently (5 times in the 250 predictors). Compared to the co-occurrence of these two terms in the training set annotations (7 times in 1609 proteins) this seems quite high, and we suggest that from the sequence perspective there is a stronger functional linkage between the two terms than was previously known. Recent experimental studies have indeed shown that ubiquitination has an important role in transcription [20]. We have not presented a detailed analysis of the annotation word combinations because it is difficult to factor out the overfitting to the training data (due to the dataset limitations discussed above), and because we would need to run many more experiments to estimate the co-occurrence frequencies accurately. However, the rudimentary analysis presented above does clearly illustrate the knowledge discovery potential of our method.

As discussed earlier, the low prediction accuracy of the evolved function predictors can be explained partly by the low-contrast training data we used. Another equally plausible explanation is that there might not always be a simple one-to-one relationship between sequence features and annotated protein functions. However, given training data of sufficient quality and quantity, we believe that novel biology can be discovered from amino acid sequences with a method such as this. We do not doubt the importance of three-dimensional structure in protein function, rather we suggest that linear motifs and features may be responsible for more biology than is currently thought. Parallels can be drawn with the recently discovered role of microRNAs in gene regulation; it is reasonable to suggest that all biological systems have evolved to use low information content components wherever possible.

Our artificial evolutionary approach for sequence feature discovery is a kind of *in silico *combinatorial screening experiment. The current implementation is non-physical and is based on regular expression matching. Although the evolution of the regular expressions is highly flexible, effective pattern discovery depends on the stepwise improvement and expansion of an initial simple pattern, with each step providing an increase in fitness. Certain patterns may therefore not be reachable unless the representation is changed (perhaps made more physical), larger populations are used, or some prior information is incorporated (such as helical wheel preprocessing). These are therefore considerations for future work.

Finally, we briefly discuss a future application of self-supervised function predictors. Our recent unpublished results show that the clustering of proteins using amino acid frequency vectors can be improved significantly by appending the binary outputs of 40 different evolved function predictors to the vectors. Most of the clusters obtained are enriched in one or more biological functions, therefore it is possible to assign/suggest functions for novel or uncharacterised proteins which fall into these clusters.

## Methods

### Datasets

The UniProt/Swiss-Prot datafile from UniProt release 1.6 (29 March 2004) was the source of sequence and annotation information for this study. It contains 146,720 entries, but we considered only eukaryotic proteins (67,392). We create a non-redundant set in a four stage approach as detailed below:

1. The UniProt/Swiss-Prot file is processed by reading from beginning to end. We only consider annotations of these types: FUNCTION, PATHWAY, PTM, CATALYTIC ACTIVITY, DEVELOPMENTAL STAGE, TISSUE SPECIFICITY, SUBCELLULAR LOCATION, MISCELLANEOUS, DOMAIN and which do not contain any of the following words: *similarity, probable, potential, possible, probably, possibly, putative, may*, or the phrases *could be, seems to be, might be*. This is our crude quality control filter for "definitive" annotations.  During processing, each FUNCTION annotation is stored in a hash table. If a protein is encountered with an already-seen FUNCTION annotation it is ignored (the Swiss-Prot curators often copy annotations from one family member to another). Proteins with no definitive FUNCTION annotation are ignored. The annotations are then split into words and a frequency count for each word is incremented. At the end of processing, a sorted list of the most frequent words is saved.

2. The list of frequent annotation words is filtered manually to remove uninformative words, such as "the" and "protein", and leave behind the 150 most frequent informative annotation words. Words indicating tissue-specific expression (such as "blood" and "muscle"), were also excluded because we did not expect this information to be present in the amino-acid sequence (perhaps wrongly). The full list is given in the Additional file 2.

3. The datafile is processed again following the procedures in step 1. This time, annotation words which are not present in the top 150 list are removed. This leaves 4908 proteins which are annotated with at least one word from the top 150. These are passed to step 4.

4. Homology-based reduction is performed using the *blastclust *program from the BLAST package version 2.2.2 [34] on the amino acid sequences of the 4908 proteins. The parameter settings for cluster inclusion are as follows: "-S 10" (minimum 10% sequence identity) and"-L 0.2" (minimum 20% alignment overlap). The final dataset of 2146 proteins is created by selecting one representative (the longest sequence) from each cluster.

The 2146 proteins are split randomly into four subsets, which we call cuts 1 to 4. In most experiments, the training set is formed from the union of cuts 1 to 3 and the testing set is cut 4. The data presented in Figures 4&5 were generated using a fourfold cross-validation procedure, where each cut of the data is used in turn as a test set. In Figure 6, the analysis is performed on the four different cuts of the data.

### Genetic programming and self-supervised learning

Genetic programming (GP) has been described by Koza [35] as an automatic method for creating computer programs using a population-based evolutionary search inspired by the natural processes of selection, mutation and recombination. We use the open source genetic programming package PerlGP [36] to evolve Perl subroutines which perform various operations on protein sequences or their annotations. The genetic material in PerlGP is a tree-like data structure, which is flattened into a piece of Perl code which is then passed to the interpreter.

The evolutionary process works on a population of 2000 individuals. At the beginning of a run, the individuals are created randomly, following a set of production rules known as a grammar. The grammar ensures that the code generated is syntactically correct. A simplified grammar is given below in Backus-Naur notation:

ROOT := sub annotation_classifier {

      my $annot = shift;

      return ANNOTBOOL;

    }

    sub sequence_classifier {

      my $seq = shift;

      return SEQNUM > SEQNUM;

    }

ANNOTBOOL := ANNOTBOOL && ANNOTBOOL |

    (ANNOTBOOL || ANNOTBOOL) |

    !(ANNOTBOOL) |

    $annot =~/ANNOTPATT/

ANNOTPATT := ANNOTPATT ANNOTPATT |

    catalyzes | golgi | mitosis ...

SEQNUM := (SEQNUM + SEQNUM) |

    (SEQNUM – SEQNUM) |

    SEQNUM * SEQNUM |

    (SEQNUM/SEQNUM) |

    abs(SEQNUM) | log(SEQNUM) |

    0 | 1 | 2 | ... | 8 | 9 |

    0.1234 | 0.7654 | ... |

    num_matches ($seq, 'SEQPATT') |

    num_matches ($seq, '^SEQPATT') |

    num_matches ($seq, 'SEQPATT$')

SEQPATT := (SEQPATT)(SEQPATT) |

    (SEQPATT)MOD | [AAS] | [^AAS]

AAS := AAS AAS | A | C | D | E ... | Y

MOD := {1,3} | {1,4} | {2,5} | ...

Because each individual is created starting at the ROOT node, the two subroutines annotation_classifier and sequence_classifier are always encoded by an individual's genotype (refer to Figure 2 for examples). The return value of annotation_classifier may be any logical combination of boolean regular expression matches (ANNOTPATT) of certain words against the annotation belonging to each sequence (a space-delimited string of annotation words). The return value of the sequence_classifier may be any arithmetic combination of numerical constants and the return values of num_matches which calculates the number of times a regular expression matches (SEQPATT) in the amino acid sequence.

The phenotype of an individual is the behaviour of these two evolved subroutines when applied to the training set data from a hard-coded loop. This loop simply passes the annotation word string for each protein in turn to annotation_classifier and stores the results in a binary vector. A second binary vector is constructed in a similar manner by passing the amino acid sequence of each protein to the sequence_classifier subroutine. In this case, however, a bit is set only if the subroutine returns true *and then also returns false for a shuffled version of the same sequence*. This forces the algorithm to discover sequence order-dependent features. The two binary vectors are then compared with each other using the correlation coefficient described by Matthews [37]. A positive correlation indicates that there are patterns in the sequences which correspond to the functions described in the annotation, and individuals with higher correlation coefficients are given a reproductive advantage within the genetic algorithm. The selection procedure follows the default parameters in PerlGP (basically this involves tournaments of 50 individuals, of which the fittest 20 reproduce to replace the least fit 20). The mutation and crossover operators obey the grammar and so always produce syntactically correct individuals. The runs were performed on machines with 2800 MHz Intel P4 processors, and were each terminated after 8 wall-clock hours had elapsed. The amino acid sequences are reshuffled every 1000 tournaments. A typical run completes around 13,000 tournaments.

Two constraints are imposed on the self-supervised learning process. First we ensure that the fraction of training set examples classified as "positive" by the annotation_classifier is between 10% and 50%. The justification for this is that we do not want the system to learn patterns that only apply to a small number of proteins. Second, our preliminary experiments showed that solutions often emerged where a simple sequence pattern was associated with a very complex combination of annotation words. Because we prefer to discover complex sequence patterns that are associated with a few annotation words we have forced the annotation_classifier to contain no more than three annotation words. In both cases these limits are implemented by "killing" any individual which fails to meet the requirements. In retrospect, we could have imposed a less arbitrary limit on the size of the annotation_classifier subroutine by simply requiring that it was smaller than the sequence_classifier subroutine.

A comprehensive listing of all the parameter settings used in the GP is not provided here, however default parameters are used extensively and all the source code and data needed to implement these experiments is provided as Additional file 3.

### "B-type" runs

The following annotation words are excluded in the "B-type" runs: "secreted", "nuclear", "membrane", "cytoplasmic", "mitochondrial", "integral", "chloroplast", "extracellular". Also, the code-generating grammar does not contain the production rules which produce anchored regular expressions (num_matches ($seq, '^SEQPATT') and num_matches ($seq, 'SEQPATT$'). Finally, the lower limit for the fraction of positive training set examples classified as "positive" by the annotation_classifier is reduced from 10% to 5% because we expect biological functions to be less prevalent than subcellular locations.

### The predictor map

In order to cluster the function predictors (in Figure 3) we calculate a binary vector for each predictor. This vector holds the outputs of the sequence_classifier subroutine which has been applied to each of the 537 test set sequences. We use a Kohonen self-organizing map (SOM) [19] to cluster the 500 vectors (250 "A-type" and 250 "B-type" predictors). The 8 × 8 rectangular SOM uses a square neighbourhood and was trained for 20 epochs using a linearly decreasing learning rate (initially 0.1) and radius (initially 4). The Euclidean distance measure is used to identify the "winning nodes" during SOM training. The predictors which map to the same SOM node are analysed in two ways, as described below.

#### Analysis of annotation_classifier subroutines

In Figure 3 we summarise the frequencies of annotation words used as targets in the evolved annotation_classifier subroutines. For example if three predictors mapped to a SOM node and their targets were "nuclear", "nuclear|dna" and "nuclear|mitosis", then these would be summarised as "3 nuclear, 1 dna, 1 mitosis". However, for reasons of space, annotation words are shown only if they are found in at least two predictors *and *in at least one quarter of the predictors. If a target contains more than one copy of the same word (e.g. "nuclear|nuclear|dna"), it is counted only once. The raw data is presented in Additional file 1.

#### Analysis of positively predicted test set sequence annotations

At each node in the SOM to which *n *predictors are mapped/clustered, two quantities, *O*_*w *_(observed fractional occurrence) and *E*_*w *_(expected fractional occurrence), are calculated for each word *w *of the 150 annotation words *W *used in our study. We define *s*_*i*,*j *_∈ {0,1} as the output of the sequence_classifier function from predictor *i *applied to sequence *j*. There are m sequences in the test set (*m *= 537), and *a*_*j*,*w *_∈ {0, 1} denotes whether sequence *j *is annotated with word *w *or not. Then, *O*_*w *_is calculated as follows:

Ow=∑i=1n∑j=1msi,j aj,w∑i=1n∑j=1m∑k∈Wsi,j aj,k
 MathType@MTEF@5@5@+=feaafiart1ev1aaatCvAUfKttLearuWrP9MDH5MBPbIqV92AaeXatLxBI9gBaebbnrfifHhDYfgasaacH8akY=wiFfYdH8Gipec8Eeeu0xXdbba9frFj0=OqFfea0dXdd9vqai=hGuQ8kuc9pgc9s8qqaq=dirpe0xb9q8qiLsFr0=vr0=vr0dc8meaabaqaciaacaGaaeqabaqabeGadaaakeaacqWGpbWtdaWgaaWcbaGaem4DaChabeaakiabg2da9maalaaabaWaaabCaeaadaaeWbqaaiabdohaZnaaBaaaleaacqWGPbqAcqGGSaalcqWGQbGAaeqaaOGaaGPaVlabdggaHnaaBaaaleaacqWGQbGAcqGGSaalcqWG3bWDaeqaaaqaaiabdQgaQjabg2da9iabigdaXaqaaiabd2gaTbqdcqGHris5aaWcbaGaemyAaKMaeyypa0JaeGymaedabaGaemOBa4ganiabggHiLdaakeaadaaeWbqaamaaqahabaWaaabuaeaacqWGZbWCdaWgaaWcbaGaemyAaKMaeiilaWIaemOAaOgabeaakiaaykW7cqWGHbqydaWgaaWcbaGaemOAaOMaeiilaWIaem4AaSgabeaaaeaacqWGRbWAcqGHiiIZcqWGxbWvaeqaniabggHiLdaaleaacqWGQbGAcqGH9aqpcqaIXaqmaeaacqWGTbqBa0GaeyyeIuoaaSqaaiabdMgaPjabg2da9iabigdaXaqaaiabd6gaUbqdcqGHris5aaaaaaa@6A75@

where the bottom term is basically the top term summed over all 150 words. The expected fraction is calculated from just the sequence annotations as:

Ew=∑j=1maj,w∑j=1m∑k∈Waj,k
 MathType@MTEF@5@5@+=feaafiart1ev1aaatCvAUfKttLearuWrP9MDH5MBPbIqV92AaeXatLxBI9gBaebbnrfifHhDYfgasaacH8akY=wiFfYdH8Gipec8Eeeu0xXdbba9frFj0=OqFfea0dXdd9vqai=hGuQ8kuc9pgc9s8qqaq=dirpe0xb9q8qiLsFr0=vr0=vr0dc8meaabaqaciaacaGaaeqabaqabeGadaaakeaacqWGfbqrdaWgaaWcbaGaem4DaChabeaakiabg2da9maalaaabaWaaabCaeaacqWGHbqydaWgaaWcbaGaemOAaOMaeiilaWIaem4DaChabeaaaeaacqWGQbGAcqGH9aqpcqaIXaqmaeaacqWGTbqBa0GaeyyeIuoaaOqaamaaqahabaWaaabCaeaacqWGHbqydaWgaaWcbaGaemOAaOMaeiilaWIaem4AaSgabeaaaeaacqWGRbWAcqGHiiIZcqWGxbWvaeaaa0GaeyyeIuoaaSqaaiabdQgaQjabg2da9iabigdaXaqaaiabd2gaTbqdcqGHris5aaaaaaa@4F00@

Annotation words are shown in the inset boxes of Figure 3 if OwEw
 MathType@MTEF@5@5@+=feaafiart1ev1aaatCvAUfKttLearuWrP9MDH5MBPbIqV92AaeXatLxBI9gBaebbnrfifHhDYfgasaacH8akY=wiFfYdH8Gipec8Eeeu0xXdbba9frFj0=OqFfea0dXdd9vqai=hGuQ8kuc9pgc9s8qqaq=dirpe0xb9q8qiLsFr0=vr0=vr0dc8meaabaqaciaacaGaaeqabaqabeGadaaakeaadaWcaaqaaiabd+eapnaaBaaaleaacqWG3bWDaeqaaaGcbaGaemyrau0aaSbaaSqaaiabdEha3bqabaaaaaaa@3246@ ≥ 3.

### Fixed-target function predictors

As discussed in the main text, GP runs were also performed using hard-coded annotation_classifier subroutines using the following targets (in the same order as the key in Figure 4): "secreted", "membrane", "integral membrane", "nuclear", "transcription|transcriptional", "dna", "inhibits|inhibit|inhibitor", "bacteria|antibacterial|gram", "cytoplasmic", "biosynthesis", "catalyzes", "mitochondrial". These experiments include the anchored regular expressions used also in "A-type" self-supervised runs. For each target, 100 independent runs were performed on machines with 2166 MHz Athlon XP processors, and were terminated after 3 wall-clock hours. For each sequence, the 100 different predictions can be combined into one "consensus prediction score" by simply taking the mean of the binary outputs.

### Per-residue analysis of predictor behaviour

Given that complex expressions are often produced by GP (see Figure 2(E) for example), it is important that we find ways to understand them more easily. We use a simple technique to estimate the contribution (positive or negative) to the final output made by each constituent regular expression in sequence_classifier. We illustrate the method with a simple example:

sub sequence_classifier {

       my $seq = shift;

       return 5 + num_matches ($seq, 'EE') >

         num_matches ($seq, ' [VIILLII]');

}

First we do some simple simplification of the regular expressions and then rearrange the whole expression to give:

       5 + num_matches ($seq, 'EE') - num_matches ($seq, ' [ILV]') > 0

We can then give each unique regular expression a symbol, *x*_*i*_, as in the following equation:

5 + *x*_1 _- *x*_2 _> 0

Then we calculate the actual number of matches made by regular expression *x*_*i *_in sequence *j*, denoted xij
 MathType@MTEF@5@5@+=feaafiart1ev1aaatCvAUfKttLearuWrP9MDH5MBPbIqV92AaeXatLxBI9gBaebbnrfifHhDYfgasaacH8akY=wiFfYdH8Gipec8Eeeu0xXdbba9frFj0=OqFfea0dXdd9vqai=hGuQ8kuc9pgc9s8qqaq=dirpe0xb9q8qiLsFr0=vr0=vr0dc8meaabaqaciaacaGaaeqabaqabeGadaaakeaacqWG4baEdaqhaaWcbaGaemyAaKgabaGaemOAaOgaaaaa@310A@ and substitute them into the equation:

5+x1j−x2j>0
 MathType@MTEF@5@5@+=feaafiart1ev1aaatCvAUfKttLearuWrP9MDH5MBPbIqV92AaeXatLxBI9gBaebbnrfifHhDYfgasaacH8akY=wiFfYdH8Gipec8Eeeu0xXdbba9frFj0=OqFfea0dXdd9vqai=hGuQ8kuc9pgc9s8qqaq=dirpe0xb9q8qiLsFr0=vr0=vr0dc8meaabaqaciaacaGaaeqabaqabeGadaaakeaacqaI1aqncqGHRaWkcqWG4baEdaqhaaWcbaGaeGymaedabaGaemOAaOgaaOGaeyOeI0IaemiEaG3aa0baaSqaaiabikdaYaqaaiabdQgaQbaakiabg6da+iabicdaWaaa@3965@

Each *x*_*i *_component in this expression is then perturbed by a small amount upwards, *p*, and downwards, *q*, and the direction in which the output changes, dij
 MathType@MTEF@5@5@+=feaafiart1ev1aaatCvAUfKttLearuWrP9MDH5MBPbIqV92AaeXatLxBI9gBaebbnrfifHhDYfgasaacH8akY=wiFfYdH8Gipec8Eeeu0xXdbba9frFj0=OqFfea0dXdd9vqai=hGuQ8kuc9pgc9s8qqaq=dirpe0xb9q8qiLsFr0=vr0=vr0dc8meaabaqaciaacaGaaeqabaqabeGadaaakeaacqWGKbazdaqhaaWcbaGaemyAaKgabaGaemOAaOgaaaaa@30E2@, is calculated. For example, for element *x*_1 _the change for sequence *j *is calculated with:

d1j=(5+(x1j+p)−x2j)−(5+(x1j−q)−x2j)
 MathType@MTEF@5@5@+=feaafiart1ev1aaatCvAUfKttLearuWrP9MDH5MBPbIqV92AaeXatLxBI9gBaebbnrfifHhDYfgasaacH8akY=wiFfYdH8Gipec8Eeeu0xXdbba9frFj0=OqFfea0dXdd9vqai=hGuQ8kuc9pgc9s8qqaq=dirpe0xb9q8qiLsFr0=vr0=vr0dc8meaabaqaciaacaGaaeqabaqabeGadaaakeaacqWGKbazdaqhaaWcbaGaeGymaedabaGaemOAaOgaaOGaeyypa0JaeiikaGIaeGynauJaey4kaSIaeiikaGIaemiEaG3aa0baaSqaaiabigdaXaqaaiabdQgaQbaakiabgUcaRiabdchaWjabcMcaPiabgkHiTiabdIha4naaDaaaleaacqaIYaGmaeaacqWGQbGAaaGccqGGPaqkcqGHsislcqGGOaakcqaI1aqncqGHRaWkcqGGOaakcqWG4baEdaqhaaWcbaGaeGymaedabaGaemOAaOgaaOGaeyOeI0IaemyCaeNaeiykaKIaeyOeI0IaemiEaG3aa0baaSqaaiabikdaYaqaaiabdQgaQbaakiabcMcaPaaa@5365@

Usually *p *= *q *= 1, but if xij
 MathType@MTEF@5@5@+=feaafiart1ev1aaatCvAUfKttLearuWrP9MDH5MBPbIqV92AaeXatLxBI9gBaebbnrfifHhDYfgasaacH8akY=wiFfYdH8Gipec8Eeeu0xXdbba9frFj0=OqFfea0dXdd9vqai=hGuQ8kuc9pgc9s8qqaq=dirpe0xb9q8qiLsFr0=vr0=vr0dc8meaabaqaciaacaGaaeqabaqabeGadaaakeaacqWG4baEdaqhaaWcbaGaemyAaKgabaGaemOAaOgaaaaa@310A@ = 0 then *q *= 0. In essence we are estimating the derivative of the output of sequence_classifier with respect to *x*_*i*_.

Then we calculate the number of times dij
 MathType@MTEF@5@5@+=feaafiart1ev1aaatCvAUfKttLearuWrP9MDH5MBPbIqV92AaeXatLxBI9gBaebbnrfifHhDYfgasaacH8akY=wiFfYdH8Gipec8Eeeu0xXdbba9frFj0=OqFfea0dXdd9vqai=hGuQ8kuc9pgc9s8qqaq=dirpe0xb9q8qiLsFr0=vr0=vr0dc8meaabaqaciaacaGaaeqabaqabeGadaaakeaacqWGKbazdaqhaaWcbaGaemyAaKgabaGaemOAaOgaaaaa@30E2@ is positive (*pos*_*i*_), zero (*zero*_*i*_) or negative (*neg*_*i*_) over all *N *sequences in the training set. To summarise the positive or negative contribution of the regular expression we calculate:

ci=posi−negiN
 MathType@MTEF@5@5@+=feaafiart1ev1aaatCvAUfKttLearuWrP9MDH5MBPbIqV92AaeXatLxBI9gBaebbnrfifHhDYfgasaacH8akY=wiFfYdH8Gipec8Eeeu0xXdbba9frFj0=OqFfea0dXdd9vqai=hGuQ8kuc9pgc9s8qqaq=dirpe0xb9q8qiLsFr0=vr0=vr0dc8meaabaqaciaacaGaaeqabaqabeGadaaakeaacqWGJbWydaWgaaWcbaGaemyAaKgabeaakiabg2da9maalaaabaGaemiCaaNaem4Ba8Maem4Cam3aaSbaaSqaaiabdMgaPbqabaGccqGHsislcqWGUbGBcqWGLbqzcqWGNbWzdaWgaaWcbaGaemyAaKgabeaaaOqaaiabd6eaobaaaaa@3E24@

Now each regular expression from an evolved predictor can be assigned a *c*_*i *_value, which ranges between -1 (always negatively influencing) to +1 (always positively influencing).

A fixed-target predictor made from 100 independently evolved classifiers can contain a few hundred regular expressions. After calculating *c_i_*for each regular expression, a protein sequence can be analysed using the following procedure:

1. assign a zero score to each residue *s*_*a *_in the sequence

2. for each of the 100 classifiers:

(a) over all constituent regular expressions *i*, calculate *sumpos *= ∑i;ci>0ci
 MathType@MTEF@5@5@+=feaafiart1ev1aaatCvAUfKttLearuWrP9MDH5MBPbIqV92AaeXatLxBI9gBaebbnrfifHhDYfgasaacH8akY=wiFfYdH8Gipec8Eeeu0xXdbba9frFj0=OqFfea0dXdd9vqai=hGuQ8kuc9pgc9s8qqaq=dirpe0xb9q8qiLsFr0=vr0=vr0dc8meaabaqaciaacaGaaeqabaqabeGadaaakeaadaaeqbqaaiabdogaJnaaBaaaleaacqWGPbqAaeqaaaqaaiabdMgaPjabcUda7iabdogaJnaaBaaameaacqWGPbqAaeqaaSGaeyOpa4JaeGimaadabeqdcqGHris5aaaa@38CB@ and *sumneg *= ∑i;ci<0ci
 MathType@MTEF@5@5@+=feaafiart1ev1aaatCvAUfKttLearuWrP9MDH5MBPbIqV92AaeXatLxBI9gBaebbnrfifHhDYfgasaacH8akY=wiFfYdH8Gipec8Eeeu0xXdbba9frFj0=OqFfea0dXdd9vqai=hGuQ8kuc9pgc9s8qqaq=dirpe0xb9q8qiLsFr0=vr0=vr0dc8meaabaqaciaacaGaaeqabaqabeGadaaakeaadaaeqbqaaiabdogaJnaaBaaaleaacqWGPbqAaeqaaaqaaiabdMgaPjabcUda7iabdogaJnaaBaaameaacqWGPbqAaeqaaSGaeyipaWJaeGimaadabeqdcqGHris5aaaa@38C7@

(b) for each regular expression *i:*

i. at all matching positions where *c*_*i *_> 0, *s*_*a *_= *s*_*a *_+ cisumpos
 MathType@MTEF@5@5@+=feaafiart1ev1aaatCvAUfKttLearuWrP9MDH5MBPbIqV92AaeXatLxBI9gBaebbnrfifHhDYfgasaacH8akY=wiFfYdH8Gipec8Eeeu0xXdbba9frFj0=OqFfea0dXdd9vqai=hGuQ8kuc9pgc9s8qqaq=dirpe0xb9q8qiLsFr0=vr0=vr0dc8meaabaqaciaacaGaaeqabaqabeGadaaakeaadaWcaaqaaiabdogaJnaaBaaaleaacqWGPbqAaeqaaaGcbaGaem4CamNaemyDauNaemyBa0MaemiCaaNaem4Ba8Maem4Camhaaaaa@3820@

ii. at all matching positions where *c*_*i *_< 0, *s*_*a *_= *s*_*a *_+ cisumneg
 MathType@MTEF@5@5@+=feaafiart1ev1aaatCvAUfKttLearuWrP9MDH5MBPbIqV92AaeXatLxBI9gBaebbnrfifHhDYfgasaacH8akY=wiFfYdH8Gipec8Eeeu0xXdbba9frFj0=OqFfea0dXdd9vqai=hGuQ8kuc9pgc9s8qqaq=dirpe0xb9q8qiLsFr0=vr0=vr0dc8meaabaqaciaacaGaaeqabaqabeGadaaakeaadaWcaaqaaiabdogaJnaaBaaaleaacqWGPbqAaeqaaaGcbaGaem4CamNaemyDauNaemyBa0MaemOBa4MaemyzauMaem4zaCgaaaaa@37F0@

This analysis was performed for the highest scoring 10% of the test set proteins (based on the consensus prediction score described above). The 500 highest scoring residues (according to *s*_*a*_) were extracted for analysis and the preparation of Figure 6. Sometimes these were single residues, and sometimes they were short fragments. The 500 most negative-scoring residues for each fixed-target function predictor were also extracted and analysed.

## Authors' contributions

MB developed the self-supervised learning approach, and performed the clustering and analysis of predictors. JH produced the training data and prototyped the self-supervised learning approach. AK developed the per-residue analysis of predictor behaviour. RMM conceived of and directed this work, studied the biological significance of the results, and wrote the text. All authors have read and approved the manuscript.

## Supplementary Material

Additional File 1 As in Figure 2 of the article, the predictors are clustered with a 8 × 8 Kohonen Self-Organising Map (SOM). In this figure, the evolved annotation word boolean expression (from the annotation_classifier subroutine) are shown in full for each of the 500 evolved function predictors. Each boolean expression is separated by a semicolon. The A-type predictors are shown with upper case to identify them. Click here for file

Additional File 2 Here we show the full list of the 150 most common annotation words after manual filtering. The filtering is performed in order to remove stopwords and words that do not contain any information about protein function. The filtered words are shown with strikethrough text. Click here for file

Additional File 3 Additional supplementary material (includes files 1 and 2); gzipped tar archive; after unpacking, please open the file brameier2005/index.html in your web browser. Click here for file

## Figures and Tables

**Table 1:** Sequence-to-function correlations.

A. All keywords						
	Experiment		Control I		Control II	
	
	mean	SE	mean	SE	mean	SE

CC on training set	0.265	0.00461	0.162	0.00226	0.209	0.00288
CC on testing set	0.112	0.00453	0.00451	0.00294	0.0664	0.00406
Top 10 keywords	secreted nuclear membrane cytoplasmic DNA biosynthesis RNA integral meiosis catalyzes					

B. Subcellular location keywords excluded						
	Experiment		Control I		Control II	
	
	mean	SE	mean	SE	mean	SE

CC on training set	0.231	0.00325	0.179	0.00240	0.213	0.00303
CC on testing set	0.0603	0.00619	0.00619	0.00276	0.0402	0.00350

Top 10 keywords	inhibits biosynthesis transcription catalyzes DNA atp bacteria stimulates transcriptional gram-negative					

This table presents the results of two experiments performed with the self-supervised learning approach. The numbers shown are the means and standard errors (SE) of correlation coefficients (CC) calculated over 250 separate runs. The CC measures the agreement, over a set of proteins, between the functional class predicted from sequence and the functional class assigned on the basis of the presence or absence of a certain combination of words in a protein's annotation. In **A**. a standard run and two controls are performed using the full vocabulary of 150 keywords. Control I simply involves the random reallocation of annotations to sequences prior to training. In Control II, sequences and annotations are correctly allocated but the amino acid sequences are randomly shuffled prior to training. In experiment **B**. the vocabulary is stripped of subcellular location keywords. The 10 most common keywords appearing in the evolved predictors are listed. In all cases the two-tailed unpaired Student's *t*-test shows a significant difference (*P *< 0.001) between the mean CCs of experiments and controls for both training and testing data. (These statistics may be calculated from the given means and standard errors, using *n *= 250.) From this one can conclude that at least some of the sequence-to-function associations learned in the training data also apply to the unrelated testing data, and therefore to proteins in general.

**Table 2:** Correlations between function predictors.

Function A	Function B	*r*_*A*,*B*_
nuclear	nuclear-copy	0.979
secreted	secreted-copy	0.964
cytoplasmic	cytoplasmic-copy	0.899

transcription	nuclear	0.860
membrane	integral	0.798
inhibits	secreted	0.780
biosynthesis	cytoplasmic	0.765
DNA	nuclear	0.737
cytoplasmic	nuclear	0.721
DNA	transcription	0.696
cytoplasmic	transcription	0.680
catalyzes	biosynthesis	0.665
antibacterial	secreted	0.643
antibacterial	inhibits	0.630
cytoplasmic	DNA	0.583
catalyzes	inhibits	-0.525
catalyzes	secreted	-0.568
inhibits	cytoplasmic	-0.598
biosynthesis	inhibits	-0.617
secreted	cytoplasmic	-0.623
biosynthesis	secreted	-0.650

Pearson's correlation coefficient, *r*_*A*,*B *_is calculated for all pairs of fixed-target predictor using the "consensus prediction scores" from test set sequences. Only predictor pairs where |*r*_*A*,*B*_| > 0.5 are shown. The strongest correlations shown at the top of the table are for "self comparisons" using duplicate predictors (trained independently with a different random seed). These indicate what "perfect" correlations would be, taking into account experimental noise. The highest non-self correlation, 0.86, is found between "nuclear" and "transcription" predictors (the raw data is shown by the blue data points in Figure 5(A)).
