# Supplementary material for: Automatic discovery of cross-family sequence features associated with protein function
Source: BMC Bioinformatics. 2006 Jan 12;7:16. doi: 10.1186/1471-2105-7-16 (PMC1395344; doi:10.1186/1471-2105-7-16)
Supplement: Additional File 1 — As in Figure 2 of the article, the predictors are clustered with a 8 × 8 Kohonen Self-Organising Map (SOM). In this figure, the evolved annotation word boolean expression (from the annotation_classifier subroutine) are shown in full for each of the 500 evolved function predictors. Each boolean expression is separated by a semicolon. The A-type predictors are shown with upper case to identify them. [file 1471-2105-7-16-S1.pdf]

	0	1	2	3	4	5	6	7
0	SECRETED ; (CHANNELS or SECRETED) ; (SECRETED or STIMULATES) ; (ELECTRON or SECRETED) ; SECRETED ; SECRETED ; SECRETED ; SECRETED ; SECRETED ; SECRETED ; SECRETED ; ((BACTERIA or SECRETED) or CHANNELS) ; SECRETED ; (SECRETED or (SECRETED or SECRETED)) ; SECRETED ; SECRETED ; SECRETED ; SECRETED ; not(not (SECRETED))	SECRETED ; SECRETED ; (GRAM-NEGATIVE or SECRETED) ; SECRETED ; SECRETED	SECRETED ; SECRETED ; ((INHIBITS or SECRETED) or SECRETED) ; MEMBRANE ; SECRETED	SECRETED ; SECRETED ; (INHIBIT or SECRETED) ; ((SECRETED or ANTIBACTERIAL) or HORMONE) ; SECRETED ; SECRETED ; (SECRETED or SECRETED) ; SECRETED ; SECRETED ; SECRETED ; SECRETED ; (SECRETED or SECRETED) ; (ELECTRON or SECRETED) ; SECRETED ; SECRETED	(gram-negative or inhibits) ; (bacteria or (secretion or inhibits)) ; inhibits ; inhibits ; (secretion or ((inhibits or electron) or electron)) ; (inhibits or hormone) ; (inhibitor or inhibits) ; (stimulates or (bacteria or inhibits)) ; (inhibits or inhibit) ; inhibits ; inhibits ; inhibits ; inhibits ; ((inhibits or bacteria) or channels) ; inhibits ; (inhibits or (calcium or stimulates)) ; ((inhibit or bacteria) or inhibits) ; (gram-negative or inhibits	SECRETED ; inhibits ; ((gram-negative or inhibits) or inhibitor) ; inhibits ; ((phosphatidylcholine or stimulates) or inhibits) ; (stimulates or (inhibit or inhibitor)) ; (gram-negative or inhibits) ; inhibits ; ((channels or inhibits) or gram-negative) ; (channels or (gram-negative or inhibits))	inhibits ; inhibits ; (bacteria or stimulates) ; not(not(inhibits)) ; ((inhibit or gram-negative) or channels) ; inhibits ; (inhibitor or (bacteria or inhibits)) ; (inhibits or inhibits) ; (inhibits or channels) ; inhibits	(hormone or (gram-negative or channels)) ; (bacteria or inhibits) ; not(regulates) and inhibits ; (gram-negative or inhibits) ; inhibits ; inhibits ; (calcium or (bacteria or inhibits)) ; (bacteria or (inhibit or inhibits)) ; inhibits and not(growth) ; (disulfide or (inhibits or stimulates)) ; (antibacterial or inhibits) ; ((secretion or bacteria) or inhibits) ; (hormone or (gram-negative or channels)) ; (bacteria or inhibits)
1	SECRETED ; (SECRETED or SECRETED) ; SECRETED ; SECRETED ; SECRETED ; (BACTERIA or SECRETED) ; SECRETED ; SECRETED ; SECRETED ; (SECRETED or SECRETED) ; SECRETED ; ((GRAM-NEGATIVE or SECRETED) or INHIBITOR) ; SECRETED ; (SECRETED or BACTERIA) ; (SECRETED or SECRETED) ; (SECRETED or DNA-BINDING)	SECRETED ; SECRETED ; SECRETED ; SECRETED	SECRETED ; SECRETED ; SECRETED ; SECRETED ; SECRETED ; SECRETED ; SECRETED ; SECRETED	((SECRETED or MITOCHONDRIAL) or INHIBITOR) ; SECRETED ; SECRETED ; SECRETED ; SECRETED ; SECRETED ; ((inhibitor or toxin) or gram-negative)	SECRETED ; SECRETED	((inhibits or inhibit) or stimulates) ; ((channels or inhibitor) or gram-negative) ; (channels or (stimulates or inhibits)) ; inhibits ; (mitosis or (dna or polymerase)) ; inhibits ; growth ; (channels or (stimulates or bacteria)) ; inhibits ; (stimulates or inhibits) ; ((antibacterial or bacteria) or channels) ; inhibits ; ((antibacterial or channels) or stimulates) ; inhibits ; (gram-negative or (inhibits or inhibits))	(gram-positive or inhibits) ; (bacteria or (inhibits or inhibitor)) ; (synthesis or replication) ; (inhibits or hormone) ; (stimulates or bacteria)	(ACTIVATE or (MATING or SECRETED)) ; (inhibits or (gram-positive or inhibitor)) ; (bacteria or (stimulates or channels)) ; inhibits ; (glutathione or phosphate) ; inhibits ; (bacteria or (stimulates or bacteria)) ; (growth or (electron or histone)) ; (secretion or (bacteria or inhibits)) ; (potassium or (secretion or bacteria))
2	(SECRETED or HYDROLYZES) ; SECRETED ; (SECRETED or (PHOSPHATIDYLCHOLINE or HYDROLYZES)) ; SECRETED ; SECRETED ; (TOXIN or SECRETED) ; (SECRETED or ACTIN) ; SECRETED ; SECRETED ; SECRETED	not(not(SECRETED)) ; SECRETED ; SECRETED	(ANTIBACTERIAL or SECRETED) ; SECRETED	((CATALYZES or MEMBRANE) or ACID) ; MEMBRANE ; MEMBRANE ; (hydrolysis or biosynthesis) ; not (nucleoside) and phosphate ; catalyzes ; ((fatty or nadph) or biosynthesis)	(CYTOPLASMIC or TRANSCRIPTIONAL) ; (INTEGRAL or (MITOSIS or GOLGI)) ; (HISTONE or (CYTOPLASMIC or UBIQUITIN)) ; atp ; (biosynthesis or (cytoplasm or diphosphate)) ; (metabolism or catalyzes) ; (catalyzes or ribosomal) ; (replication or (biosynthesis or catalyzes)) ; dna ; biosynthesis ; catalyzes ; catalyzes	((MITOTIC or DNA) or CHLOROPLAST) ; (MITOCHONDRIAL or ((TRANSCRIPTION or ATP) or INTERACTS)) ; (MEMBRANE or (INHIBIT or CONTROL)) ; (diphosphate or reticulum) ; (rna or biosynthesis) ; biosynthesis ; (catalyzes or coa) ; catalyzes ; (uptake or (udp or (collagen or biosynthesis))) ; biosynthesis ; biosynthesis ; atp ; (atp or (biosynthesis or reticulum)) ; biosynthesis ; (biosynthesis or biosynthesis) ; not(regulation) and atp ; atp ; biosynthesis	((receptor or protease) or oxidized) ; ((acid or catalyzes) or hydrolysis) ; (receptor or adhesion) ; transcription ; not(acid) and catalyzes ; transcription ; ((transcription or chromatin) or dna-binding) ; (polymerase or (repressor or catalyzes)) ; transcription ; (mitotic or dna) ; atp ; (dna or (interacts or promoter)) ; (receptor or (protease or oxidized)) ; biosynthesis ; (development or (acids or (rna or transcriptional))) ; (catalyzes or spindle) ; ((transcriptional or chromatin) or transcription) ; catalyzes ; ((transcription or chromosome) or activator) ; (transcription or (apoptosis or electron)) ; (response or (control or catalyzes)) ; transcription ; transcription ; phosphate ; ((regulates or atp) or biosynthesis)	(development or transcription) ; transcription ; ((development or initiation) or dna) ; (development or (transcription or interacts)) ; transcription ; transcription ; ((transcription or kinase) or regulator) ; (((development or transcription) or response) or spindle) ; ((transcription or kinase) or polymerase) ; ((interacts or repair) or transcription) ; (repair or (polymerase or (transcription or development))) ; (((dna or dna) or chromosome) or mitosis) ; (dna or (transcription or kinase)) ; transcription ; (transcription or dna) ; transcription ; (transcriptional or polymerase) ; ((transcription or cleaves) or development)
3	SECRETED ; (ENZYME or (SURFACE or INTEGRAL))		(MEMBRANE or SIGNAL) ; (MITOCHONDRIAL or (REPAIR or TRANSCRIPTION)) ; (ADP or MITOCHONDRIAL) ; (TRANSFER or (INTEGRAL or ATP)) ; (biosynthesis or (fatty or development)) ; ((biosynthesis or diphosphate) or nadph)	(SULFATE or (PHOSPHATE or DNA)) ; (REPAIR or CATALYZES) ; biosynthesis ; catalyzes ; (biosynthesis or cytoplasm) ; ((biosynthesis or (phosphate or transcription)) or signaling) ; ((transcriptional or synthesis) or phosphorylated) ; catalyzes ; (glucose or (biosynthesis or transcriptional)) ; ((biosynthesis or biosynthesis) or chromosome) ; (biosynthesis or (meiosis or catalyzes))	biosynthesis ; biosynthesis ; (repression or ((biosynthesis or mitosis) or golgi)) ; biosynthesis ; biosynthesis ; biosynthesis ; biosynthesis ; catalyzes ; (biosynthesis or (metabolism or transcription)) ; biosynthesis	(RNA or NUCLEAR) ; (NUCLEAR or VACUOLAR) ; MEMBRANE ; atp ; (transcriptional or (phosphorylated or mrna)) ; ((mrna or transcription) or transcriptional) ; synthesis ; (reticulum or (fatty or cleaves)) ; ((amp or rna) or biosynthesis) ; (cycle or (golgi or biosynthesis)) ; (catalyzes or (udp or cycle)) ; catalyzes ; biosynthesis ; biosynthesis	MEMBRANE ; CYTOPLASMIC ; ((((biosynthesis or phosphorylation) or cleavage) or glycosylation) or surface) ; (udp or catalyzes) ; (golgi or (udp or biosynthesis)) ; (actin or catalyzes)	((RECEPTOR or INTEGRAL) or ATP) ; (MEMBRANE or (SPLICING or GROWTH)) ; biosynthesis ; transcription ; (development or adp) ; ((hydrolysis or catalyzes) or acid) ; (biosynthesis or repression) ; atp ; (transcription or (rna or biosynthesis)) ; ((meiosis or transcriptional) or mrna) ; (nucleoside or (glucose or catalyzes))
4	MEMBRANE ; ((INTEGRAL or COA) or BIOSYNTHESIS) ; (SYNTHESIS or MEMBRANE) ; (PHOSPHORYLATION or (DISULFIDE or MEMBRANE)) ; MEMBRANE ; ((UPTAKE or (INTEGRAL or COA)) or BIOSYNTHESIS)	CYTOPLASMIC ; (((TRANSCRIPTIONAL or DNA) or MITOCHONDRIAL) or PHOSPHATE) ; ((DNA or BIOSYNTHESIS) or CYTOPLASMIC)	(PHOSPHATE or (REGULATES or INTEGRAL)) ; MEMBRANE ; (0 or ((MEMBRANE or MITOCHONDRIAL) or DEVELOPMENT)) ; (RESPONSE or (CATALYZES or ACID)) ; (CYTOPLASMIC or BIOSYNTHESIS) ; (CATALYZES or (CATALYZES or PHOSPHATE)) ; MEMBRANE ; (RETICULUM or CATALYZES) ; (golgi or biosynthesis) ; not(not(not(not(not (biosynthesis))))))	(NUCLEAR or MATING) ; (BIOSYNTHESIS or NUCLEAR) ; (MITOCHONDRIAL or INTEGRAL) ; (ATP or (MEMBRANE or RNA)) ; (biosynthesis or dna) ; (atp or biosynthesis)	((catalyzes or golgi) or cycle) ; biosynthesis ; (0 or (phosphorylation or (mrna or replication))) ; dna	NUCLEAR ; transcription ; biosynthesis ; ((transcription or apoptosis) or biosynthesis) ; (mrna or (dna or transcription)) ; transcription ; transcription ; (control or (diphosphate or dna)) ; (development or transcriptional)	(RNA or (METABOLISM or MEMBRANE)) ; ((CATALYZES or CATALYZES) or GROWTH) ; (promoters or (biosynthesis or golgi)) ; ((transcriptional or mrna) or atp) ; catalyzes	(DIPHOSPHATE or MEMBRANE) ; (ATP or (CYTOPLASMIC or PHOSPHORYLATED)) ; NUCLEAR ; (BIOSYNTHESIS or (NUCLEAR or CYTOPLASMIC)) ; (RNA or NUCLEAR) ; dna ; (biosynthesis or mrna)
5	((BIOSYNTHESIS or MEMBRANE) or UPTAKE) ; (DEVELOPMENT or (BIOSYNTHESIS or MEMBRANE)) ; (MRNA or (NUCLEAR or MEMBRANE)) ; MEMBRANE ; ((MEMBRANE or ATP) or GLUCOSE) ; ((BIOSYNTHESIS or CYTOPLASMIC) or DEGRADATION)	MEMBRANE	CYTOPLASMIC ; ((INTERACTION or DNA) or REGULATES) ; CYTOPLASMIC	NUCLEAR ; (NUCLEAR or MITOSIS) ; ((promoter or dna) or phosphorylated)	(DEVELOPMENT or (NUCLEAR or REGULATOR)) ; (INITIATION or (VESICLES or NUCLEAR)) ; (NUCLEAR or IMPORT) ; (NUCLEAR or RNA) ; NUCLEAR ; (VESICLES or (REGULATOR or NUCLEAR)) ; ((GTP or REGULATOR) or NUCLEAR) ; NUCLEAR ; NUCLEAR	NUCLEAR ; transcription ; ((promoters or transcription) or activator) ; ((rna or transcription) or regulates) ; ((activator or (promoters or transcription)) or rna) ; ((activator or transcription) or promoters) ; transcription and not(nucleus) ; transcription ; (mitotic or (transcription or transcriptional)) ; ((activator or transcription) or histone) ; (activator or (repression or transcription)) ; transcription	(UDP or (NUCLEAR or ADP)) ; (NUCLEAR or (MITOSIS or ATPASE)) ; NUCLEAR ; CYTOPLASMIC ; transcription	(CYTOPLASMIC or (CATALYZES or HYDROLYSIS)) ; (MEIOSIS or (CYTOPLASMIC or RNA)) ; (CYTOPLASMIC or (RESISTANCE or GOLGI)) ; (NADP or NUCLEAR) ; ((phosphate or meiosis) or atp) ; (mitosis or (tyrosine or replication)) ; (biosynthesis or ((transcriptional or dna) or phosphorylation)) ; (golgi or (plasma or interacts))
6	((REGULATORY or MEMBRANE) or DEVELOPMENT) ; ((BIOSYNTHESIS or (CYTOPLASMIC or MEMBRANE)) or MITOCHONDRIAL) ; ((MRNA or CONTROL) or INTEGRAL) ; ((DIPHOSPHATE or CYTOPLASMIC) or MEMBRANE) ; ((BIOSYNTHESIS or COA) or MEMBRANE) ; (CYTOPLASMIC or MEMBRANE)	(METABOLISM or (CYTOPLASMIC or NUCLEAR)) ; ((CHLOROPLAST or DNA) or ATP) ; (BIOSYNTHESIS or (FATTY or MEMBRANE)) ; ((CYTOPLASMIC or NUCLEAR) or PHOSPHORYLATED)	(DNA or NUCLEAR) ; ((DNA or KINASE) or NUCLEAR) ; NUCLEAR ; ((NUCLEAR or KINASE) or UBIQUITIN)	NUCLEAR ; NUCLEAR ; (NUCLEAR or INITIATION) ; NUCLEAR ; NUCLEAR and not(MEIOSIS) ; NUCLEAR ; NUCLEAR	NUCLEAR ; (NUCLEAR or CYTOPLASMIC) ; (NUCLEAR or (REGULATION or SIGNALING)) ; (((NUCLEAR or REGULATION) or SIGNALING) or REPLICATION)	NUCLEAR ; dna ; (dna or chromosome) ; (transcription or transcriptional)	NUCLEAR ; ((biosynthesis or transcription) or udp)	(INTEGRAL or (NUCLEAR or BIOSYNTHESIS)) ; ((PHOSPHORYLATED or NUCLEAR) or BIOSYNTHESIS) ; NUCLEAR
7	((CYTOPLASMIC or NUCLEAR) or DNA) ; (NUCLEAR or CYTOPLASMIC) ; ((CYTOPLASMIC or NUCLEAR) or BIOSYNTHESIS) ; NUCLEAR	(NUCLEAR or DNA) ; ((NUCLEAR or GTP) or RNA)	(RNA or NUCLEAR) ; ((DNA or MITOTIC) or NUCLEAR) ; (MEIOSIS or (NUCLEAR or DNA)) ; NUCLEAR ; (CHROMATIN or (RNA or NUCLEAR)) ; (MITOSIS or (NUCLEAR or MEIOSIS)) ; NUCLEAR ; (RNA or NUCLEAR) ; ((NUCLEAR or RNA) or NUCLEAR) ; NUCLEAR	(NUCLEAR or (MEIOSIS or NUCLEAR))	NUCLEAR ; (REPRESSION or (DNA or NUCLEAR)) ; (NUCLEAR or DNA) ; ((transcription or dna) or repression) ; (transcription or dna)	(DNA or (NUCLEAR or REPRESSION)) ; ((CHROMATIN or NUCLEAR) or DNA) ; (DNA or NUCLEAR) ; ((NUCLEAR or DNA) or PROMOTERS) ; (DNA or (UBIQUITIN or NUCLEAR)) ; (REPRESSION or (NUCLEAR or DNA)) ; ((NUCLEAR or DNA) or UBIQUITIN) ; ((rna or dna) or transcription) ; dna ; ((transcription or repression) or dna) ; ((transcription or chromatin) or dna) ; (chromosome or dna) ; (repression or dna) ; dna ; ((transcriptional or transcription) or dna) ; (transcription or (repression or (transcriptional or dna)))		(REGULATOR or (NUCLEAR or REGULATION)) ; ((UBIQUITIN or NUCLEAR) or BIOSYNTHESIS) ; (BIOSYNTHESIS or NUCLEAR) ; (PROMOTER or (NUCLEAR or MITOSIS)) ; ((MEIOSIS or NUCLEAR) or TRANSCRIPTION) ; ((NUCLEAR or MEIOSIS) or TRANSCRIPTION) ; NUCLEAR ; ((NUCLEAR or TRANSCRIPTION) or MEIOSIS) ; (REGULATION or NUCLEAR) ; (REGULATION or (PHOSPHATE or NUCLEAR)) ; NUCLEAR ; not(not (NUCLEAR)) ; (REGULATION or NUCLEAR) ; (UBIQUITIN or NUCLEAR)
