# Supplementary material for: Automatic discovery of cross-family sequence features associated with protein function
Source: BMC Bioinformatics. 2006 Jan 12;7:16. doi: 10.1186/1471-2105-7-16 (PMC1395344; doi:10.1186/1471-2105-7-16)
Supplement: Additional File 2 — Here we show the full list of the 150 most common annotation words after manual filtering. The filtering is performed in order to remove stopwords and words that do not contain any information about protein function. The filtered words are shown with strikethrough text. [file 1471-2105-7-16-S2.html]

|  |
| --- |
| ~~the~~ |
| ~~of~~ |
| ~~and~~ |
| ~~in~~ |
|  |
| ~~a~~ |
| ~~to~~ |
| ~~is~~ |
| ~~for~~ |
| ~~protein~~ |
| ~~by~~ |
| ~~with~~ |
| **membrane** |
| ~~activity~~ |
| ~~as~~ |
| **nuclear** |
| ~~involved~~ |
| **secreted** |
| ~~that~~ |
| ~~binds~~ |
| ~~an~~ |
| ~~this~~ |
| ~~it~~ |
| ~~cell~~ |
| ~~required~~ |
| **receptor** |
| ~~from~~ |
| ~~are~~ |
| ~~proteins~~ |
| ~~role~~ |
| ~~on~~ |
| ~~has~~ |
| ~~at~~ |
| ~~which~~ |
| ~~complex~~ |
| ~~or~~ |
| **dna** |
| **cytoplasmic** |
| **integral** |
| ~~cells~~ |
| ~~also~~ |
| ~~not~~ |
| ~~h(2)o~~ |
| ~~binding~~ |
| ~~factor~~ |
| **catalyzes** |
| ~~type~~ |
| **acid** |
| **transcription** |
| **atp** |
| ~~into~~ |
| ~~i~~ |
| ~~domain~~ |
| **inhibits** |
| **phosphate** |
| **biosynthesis** |
| ~~essential~~ |
| ~~during~~ |
| ~~acts~~ |
| ~~its~~ |
| **mitochondrial** |
| ~~plays~~ |
| ~~but~~ |
| ~~formation~~ |
| ~~both~~ |
| **adp** |
| ~~genes~~ |
| **activation** |
| ~~pathway~~ |
| **enzyme** |
| **calcium** |
| ~~function~~ |
| **transport** |
| **growth** |
| ~~component~~ |
| ~~against~~ |
| ~~can~~ |
| **development** |
| ~~form~~ |
| ~~step~~ |
| **transcriptional** |
| ~~2~~ |
| **tyrosine** |
| ~~residues~~ |
| **kinase** |
| ~~subunit~~ |
| **rna** |
| ~~ii~~ |
| ~~other~~ |
| ~~two~~ |
| ~~system~~ |
| ~~gene~~ |
| **regulation** |
| ~~sequence~~ |
| **response** |
| ~~be~~ |
| **mediates** |
| **channels** |
| **synthesis** |
| ~~active~~ |
| ~~specific~~ |
| **fatty** |
| ~~expression~~ |
| ~~peptide~~ |
| **hydrolysis** |
| ~~important~~ |
| ~~functions~~ |
| **phosphorylation** |
| ~~through~~ |
| **cleavage** |
| **reticulum** |
| **diphosphate** |
| ~~chain~~ |
| ~~endoplasmic~~ |
| ~~one~~ |
| **receptors** |
| **inhibitor** |
| ~~site~~ |
| **release** |
| ~~high~~ |
| **phosphorylated** |
| ~~responsible~~ |
| ~~isoform~~ |
| **activates** |
| **activated** |
| ~~such~~ |
| ~~between~~ |
| **plasma** |
| ~~bind~~ |
| ~~does~~ |
| ~~major~~ |
| ~~g~~ |
| **signal** |
| **acids** |
| ~~specifically~~ |
| **cycle** |
| **interacts** |
| ~~region~~ |
| ~~affinity~~ |
| ~~their~~ |
| ~~1~~ |
| ~~muscle~~ |
| ~~b~~ |
| ~~c-terminal~~ |
| **interaction** |
| **potassium** |
| **extracellular** |
| ~~c~~ |
| **matrix** |
| ~~alpha~~ |
| **control** |
| ~~substrates~~ |
| **signaling** |
| **channel** |
| **degradation** |
| **golgi** |
| **polymerase** |
| ~~o(2~~ |
| ~~could~~ |
| ~~forms~~ |
| ~~second~~ |
| ~~found~~ |
| ~~first~~ |
| **differentiation** |
| ~~when~~ |
| ~~mediated~~ |
| ~~motif~~ |
| ~~3~~ |
| ~~part~~ |
| ~~via~~ |
| **ubiquitin** |
| **regulates** |
| ~~induces~~ |
| **activator** |
| ~~known~~ |
| ~~including~~ |
| **stimulates** |
| ~~act~~ |
| ~~assembly~~ |
| ~~cellular~~ |
| ~~play~~ |
| **actin** |
| ~~domains~~ |
| ~~associated~~ |
| ~~contains~~ |
| **regulatory** |
| **intracellular** |
| ~~n-terminal~~ |
| ~~potent~~ |
| ~~residue~~ |
| ~~amino~~ |
| **protease** |
| ~~precursor~~ |
| **hormone** |
| **bacteria** |
| ~~vitro~~ |
| ~~than~~ |
| **regulator** |
| **surface** |
| ~~necessary~~ |
| ~~substrate~~ |
| **histone** |
| ~~have~~ |
| ~~reduced~~ |
| ~~no~~ |
| ~~early~~ |
| **promoter** |
| ~~bond~~ |
| ~~variety~~ |
| ~~three~~ |
| **metabolism** |
| **nadph** |
| ~~n~~ |
| ~~sites~~ |
| ~~appears~~ |
| **serine** |
| **activate** |
| ~~specificity~~ |
| ~~only~~ |
| ~~seems~~ |
| ~~central~~ |
| **repair** |
| ~~promotes~~ |
| ~~element~~ |
| ~~all~~ |
| **phosphoprotein** |
| ~~small~~ |
| ~~well~~ |
| ~~low~~ |
| **nucleus** |
| ~~group~~ |
| ~~n-terminus~~ |
| ~~beta~~ |
| ~~thought~~ |
| **mitosis** |
| ~~ions~~ |
| ~~terminal~~ |
| ~~activities~~ |
| ~~thereby~~ |
| **nadp** |
| **cleaves** |
| ~~bonds~~ |
| ~~normal~~ |
| ~~causes~~ |
| ~~present~~ |
| ~~containing~~ |
| **proliferation** |
| ~~inner~~ |
| **sodium** |
| **cyclase** |
| **gram-positive** |
| ~~presence~~ |
| ~~several~~ |
| ~~action~~ |
| ~~ph~~ |
| ~~processing~~ |
| ~~various~~ |
| ~~effect~~ |
| **chloroplast** |
| **membrane-bound** |
| **antibacterial** |
| **hydrolyzes** |
| ~~blocked~~ |
| **mrna** |
| **glucose** |
| **replication** |
| ~~shows~~ |
| ~~different~~ |
| ~~complexes~~ |
| ~~negative~~ |
| ~~trypsin~~ |
| **membranes** |
| **apoptosis** |
| **neuronal** |
| ~~within~~ |
| **flavoprotein** |
| **inhibit** |
| ~~participates~~ |
| **amp** |
| **adhesion** |
| ~~recognizes~~ |
| ~~chains~~ |
| ~~conversion~~ |
| **import** |
| **spindle** |
| **cytoplasm** |
| **sulfate** |
| **toxin** |
| **vesicles** |
| ~~thus~~ |
| ~~position~~ |
| ~~embryonic~~ |
| **secretion** |
| **transduction** |
| ~~after~~ |
| ~~process~~ |
| ~~number~~ |
| **catalytic** |
| **disulfide** |
| ~~they~~ |
| ~~exhibits~~ |
| ~~blood~~ |
| ~~production~~ |
| ~~reaction~~ |
| **gtp** |
| **nad** |
| ~~some~~ |
| **glycosylation** |
| **initiation** |
| ~~cysteine~~ |
| ~~association~~ |
| **dna-binding** |
| **glutathione** |
| **atpase** |
| ~~peptides~~ |
| ~~these~~ |
| **transfer** |
| ~~molecule~~ |
| **ribosomal** |
| **mating** |
| ~~manner~~ |
| ~~more~~ |
| **chromatin** |
| ~~many~~ |
| **splicing** |
| ~~anion~~ |
| ~~exchange~~ |
| **gram-negative** |
| **chromosome** |
| **oxidized** |
| ~~repeats~~ |
| ~~inhibition~~ |
| ~~human~~ |
| ~~subunits~~ |
| ~~and/or~~ |
| ~~there~~ |
| ~~target~~ |
| **coa** |
| **mitotic** |
| **nadh** |
| ~~converts~~ |
| ~~directly~~ |
| ~~groups~~ |
| ~~wall~~ |
| **cytochrome** |
| **promoters** |
| ~~phase~~ |
| **resistance** |
| ~~preferentially~~ |
| ~~highly~~ |
| ~~tissues~~ |
| ~~blocks~~ |
| **calcium-dependent** |
| ~~soluble~~ |
| ~~molecules~~ |
| ~~structure~~ |
| ~~body~~ |
| ~~key~~ |
| **integrin** |
| **phosphorylates** |
| **phosphatidylcholine** |
| ~~composed~~ |
| ~~large~~ |
| ~~yeast~~ |
| ~~mediate~~ |
| ~~where~~ |
| ~~plant~~ |
| ~~most~~ |
| **inactivation** |
| ~~core~~ |
| ~~processes~~ |
| **repression** |
| ~~maintenance~~ |
| **collagen** |
| **vacuolar** |
| ~~neurons~~ |
| **acceptor** |
| **nucleoside** |
| **messenger** |
| ~~conjugation~~ |
| **udp** |
| ~~iii~~ |
| ~~co(2~~ |
| ~~controls~~ |
| ~~implicated~~ |
| **uptake** |
| **division** |
| ~~components~~ |
| ~~ion~~ |
| ~~nervous~~ |
| ~~elements~~ |
| ~~ligand~~ |
| ~~localization~~ |
| ~~level~~ |
| ~~4~~ |
| ~~possesses~~ |
| ~~family~~ |
| **electron** |
| ~~consensus~~ |
| ~~factors~~ |
| ~~pore~~ |
| ~~death~~ |
| **repressor** |
| ~~then~~ |
| ~~broad~~ |
| ~~might~~ |
| ~~class~~ |
| **proteolytic** |
| ~~basic~~ |
| ~~proper~~ |
| ~~transition~~ |
| ~~higher~~ |
| **meiosis** |
