# Supplementary material for: Automatic discovery of cross-family sequence features associated with protein function
Source: BMC Bioinformatics. 2006 Jan 12;7:16. doi: 10.1186/1471-2105-7-16 (PMC1395344; doi:10.1186/1471-2105-7-16)
Supplement: Additional File 3 — Additional supplementary material (includes files 1 and 2); gzipped tar archive; after unpacking, please open the file brameier2005/index.html in your web browser. [file 1471-2105-7-16-S3.gz › brameier2005/index.html]

Supplementary information for "Automatic Discovery of Cross-Family Sequence Features Associated with Protein Function"


|  |
| --- |
| Supplementary information for Automatic Discovery of Cross-Family Sequence Features Associated with Protein FunctionMarkus Brameier, Josien Haan, Andrea Krings and Robert M. MacCallum  Stockholm Bioinformatics Center, Stockholm University, 106-91 Stockholm, Sweden  Jump to Supplementary Figure A, Supplementary Table A, or the Supplementary Material. Supplementary Figure AView/downloadAvailable in HTML and PDF formats. Please note that the PDF document may have to be rescaled for printing.DescriptionAs in Figure 2 of the article, the predictors are clustered with a 8 x 8 Kohonen Self-Organising Map (SOM). In this figure, the evolved annotation word boolean expression (from the annotation\_classifier subroutine) are shown in full for each of the 500 evolved function predictors. Each boolean expression is separated by a semicolon. The A-type predictors are shown with upper case to identify them.Supplementary Table AViewAvailable in HTML format.DescriptionHere we show the full list of the 150 most common annotation words after manual filtering. The filtering is performed in order to remove stopwords and words that do not contain any information about protein function. The filtered words are shown with ~~strikethrough~~ text.Supplementary MaterialMatthews Correlation CoefficientCC = (tp\*tn - fn\*fp) / sqrt( (tn+fn)(tn+fp)(tp+fn)(tp+fp) )Training and testing dataWhere four-fold cross-validation was performed, each of the following "cuts" of the data was used as a test set, while the other three were used as training data. In all other cases, the "training set" is the concatenation of cuts 1 to 3 and the "test set" is cut 4.  - Cut 1- Cut 2- Cut 3- Cut 4  Datafile description: column 1 contains the UniProt/Swiss-Prot ID, column 2 contains the UniProt/Swiss-Prot AC, column 3 contains the amino acid sequence, columns 4 and onwards contain the annotation words.Running it yourself with PerlGPHere are some guidelines if you would like to try the self-supervised learning yourself.  1. Install PerlGP and get to the    stage where you can run one or two of the demos and know how to look    at the results they produce. At present, support can only be given    for Unix-like operating systems (e.g. Linux, Mac OS X), but it may    work on others.- Install PDL, if your Perl installation doesn't already have it.- Create a new directory called "funcpred" (for example), download        this tar.gz file and unpack it so        that you create a subdirectory "funcpred/funcpred-demo".- Create a new subdirectory "funcpred/data" and in this directory,          concatenate three of the datafiles (cuts 1 to 4 above) into "training.dat" and rename          remaining one as "testing.dat".- Now you should be able to start the self-supervised learning with: # change directory to the "experiment directory"             cd funcpred/funcpred-demo                          # quickly check that the data files are where they should be             ls ../data/training.dat ../data/testing.dat                          # clean out any old data for this experiment             perlgp-wipe-expt.pl                          # this program shows you what a random program looks like             # (like those in the initial population)             perlgp-rand-prog.pl                          # start the run so that it restarts if there are any unavoidable crashes             perlgp-run.pl -loop             (ignore the warnings about divide by zero and log(x≤0))  Please contact Bob MacCallum if you have any problems or questions. |
